# Role of Adenosine A1 Receptor in Sleep Deprivation-Induced Neuroinflammation: Insights on Rapid Eye Movement Sleep and Fear Extinction Memory Recall in Rats

**DOI:** 10.7759/cureus.75926

**Published:** 2024-12-18

**Authors:** Bhanuteja Thondala, Garima Chauhan, Harsh Pawar, Koushik Ray, Monika Sharma, Neha Yadav, Sanjeev Kumar, Krishna Kishore, Usha Panjwani

**Affiliations:** 1 Department of Human Factors Engineering and Military Ergonomics, Defence Institute of Physiology and Allied Sciences, Delhi, IND; 2 Physiology, All India Institute of Medical Sciences, New Delhi, New Delhi, IND; 3 School of Allied Health Sciences, Manav Rachna International Institute of Research and Studies, Faridabad, IND

**Keywords:** adenosine a1 receptor, electroencephalogram, fear extinction memory recall, neuroinflammation, power spectral density, recovery sleep, sleep deprivation, synaptic plasticity, toll-like receptor 4

## Abstract

Introduction: Sleep deprivation (SD), stemming from a myriad of aetiologies, is a prevalent health condition frequently overlooked. It typically impairs memory consolidation and synaptic plasticity, potentially through neuroinflammatory mechanisms and adenosinergic signalling. It is still unclear whether the adenosine A1 receptor (A1R) modulates SD-induced neurological deficits in the hippocampus.

Objectives: This study aims to evaluate the effects of SD on fear extinction memory recall and emotional behaviour in male Sprague Dawley rats; to investigate the role of A1R antagonism by the administration of 8-cyclopentyltheophylline (8-CPT), an A1R antagonist during 48-hour SD in mitigating neuroinflammation and synaptic plasticity deficits induced by SD; and to assess changes in hippocampal neurogenesis, neuronal cell death, and sleep architecture in response to A1R antagonism during SD.

Methods: A total of 39 animals were used in the study, and they were divided into three experimental groups: 1) cage control (CC; n = 13); 2) SD for 48 hours (SD; n = 13); 3) SD for 48 hours+ 8-CPT (20 mg/kg/day in 20% DMSO divided into two doses, morning and evening, i.p.; n = 13). ‘n’ refers to the sample size/number of animals in each group. Rats were subjected to SD after cued fear extinction training for 48 hours followed by fear extinction memory recall test, anxious-depressive-like behaviours by open field test (OFT), sucrose preference test, and forced swim test (FST). Levels of adenosine in the hippocampus were quantified by high-performance liquid chromatography. Protein levels of interleukin-6 (IL-6) and IL-10 were quantified by enzyme-linked immunosorbent assay (ELISA). Expression levels of proteins and genes of interest were analysed using immunohistochemistry and real-time polymerase chain reaction (RT-PCR), respectively. Sleep architecture was assessed by recording electroencephalography (EEG), electromyography, and electrooculography from rats.

Results: Administration of CPT during SD reversed extinction recall impairments (p = 0.01), improved line crossings in OFT, sucrose preference (p < 0.01), and reduced immobility during the FST (p < 0.01). Immunohistochemical analysis of DG, CA3, and CA1 regions of the hippocampus revealed a significant upregulation of A1R expression in the SD and SD+CPT groups (p < 0.001, n = 5). Expression of post-synaptic density protein (PSD-95) and synaptophysin increased and a marked reduction in the Toll-like receptor-4 (TLR-4) expression in activated microglia in the SD+CPT group. 8-CPT partially restored SD-induced decline in serotonin and brain-derived neurotrophic factor. SD-induced neuronal apoptosis through caspase-3 and the P-p38 mitogen-activated protein kinase pathway was partially reversed by 8-CPT. RT-PCR results showed that A1R antagonism attenuated gene expression of pro-inflammatory cytokines (IL-1β, TNFα, p-NFκB s536, and IL-6) and increased anti-inflammatory cytokines (IL-1ra, IL-4, IL-10, IL-11, and IL-13) during SD. EEG recordings revealed that A1R antagonism increased REM sleep without affecting non-REM sleep during SD, leaving rebound sleep unaffected.

Conclusion: These findings highlight the role of A1R antagonism in restoring fear extinction memory recall, synaptic plasticity, adult neurogenesis, neuronal cell death, and attenuating neuroinflammation during SD, paving the way for the further exploration of its therapeutic potential in sleep-related cognitive deficits.

## Introduction

Sleep is an evolutionarily conserved phenomenon crucial for numerous physiological processes, yet its exact biological function remains elusive; however, it is well-established that loss of sleep can lead to low-grade neuroinflammation, contributing to cardiovascular diseases, immune dysfunction, and metabolic disorders. Sleep homeostasis is regulated by accumulating sleep pressure marked by key somnogens such as adenosine, an extracellular by-product of cellular metabolism to mediate homeostasis [1]. The role of adenosine signalling and adenosine A1 receptor (A1R) in modulating neurological processes during sleep deprivation (SD) has garnered increasing interest. Activation of A1R by adenosine has been reported to disrupt the acquisition of contextual fear conditioning in rats [2], and downregulation of the PKA pathway contributes to memory impairment following SD [3]. Experimental data suggest that A1R expression increases during chronic SD in several brain regions, including the hippocampus [4]. However, there is no clear evidence of the impact of SD and A1R activation/inhibition on fear extinction memory.

Interestingly, A1R knockout mice exhibit impaired synaptic plasticity [5] and depressive-like behaviours showing resistance to the anti-depressant effects of SD. Contrarily, the overexpression of A1R in the hippocampus also causes depressive-like behaviour [6], emphasizing the role of A1R in SD-induced alterations in memory and emotional states [7]. It was also demonstrated that A1R blockade in the hippocampus by 8-cyclopentyltheophylline (8-CPT) rescued synaptic plasticity deficits induced by six-hour SD [8]. These conflicting reports intrigued us to investigate whether the blockade of A1R signalling by 8-CPT modulates the 48-hour SD-induced behavioural and synaptic plasticity deficits in the hippocampus. SD regulates memory and anxious-depressive-like behaviours in animal models, causing deficits in memory [9], attention [10], and circadian rhythms [11] by augmenting the activation of glial cells and causing low-grade neuroinflammation, resulting in the altered microenvironment of brain regions [12]. Disruption of sleep homeostasis leads to neuroinflammation via the activation of Toll-like receptors (TLRs) in the hippocampus, which recognises damage-associated molecular patterns (DAMPs) and regulates fear memories [13] and activated microglia in neonatal rat brain following hypoxia. The role of A1R in modulating TLR4-mediated microglial activation and cytokines during SD for alleviating neuroinflammation and fear memories is underexplored.

A1Rs are crucial in mediating inter-individual differences in resilience to SD. Elevated A1R availability in humans represents increased resilience to the adverse effects of SD, as compared to individuals with lower A1R availability [14]. This suggests a protective role of higher A1R availability against SD-induced deficits. Accordingly, we conducted an investigation to elucidate the relationship between A1R expression and sleep architecture, aiming to enhance our understanding of A1R's role in dysregulated sleep architecture. Our prior work characterised the deterioration of spatial reference memory and neurogenesis during SD, ameliorated by A1R antagonism [15]. In the present study, we studied the role of A1R antagonism in mitigating SD-induced TLR4-mediated microglial activation, rescuing synaptic plasticity, fear extinction memory recall, anxious-depressive-like states, and rebound sleep architecture. However, there is a lack of studies investigating the association between SD-induced TLR4-mediated microglial activation and regulation of neuroinflammation, adult neurogenesis, and fear extinction memory recall via adenosine A1 receptors. In the present study, we hypothesise that 48-hour SD induces neuroinflammation and cognitive impairments via A1R-mediated pathways and that pharmacological inhibition of A1R with 8-CPT can mitigate these deficits by reducing TLR4 expression, rescuing synaptic plasticity, fear extinction memory recall, adult neurogenesis, cell death, and rebound sleep architecture. This study aims to evaluate the effects of SD on fear extinction memory recall and emotional behaviour in rats. We investigated the role of A1R antagonism in mitigating neuroinflammation and synaptic plasticity deficits induced by SD. Furthermore, an assessment of hippocampal neurogenesis, neuronal cell death, and sleep architecture in response to A1R antagonism during SD was done.

We aimed to elucidate mechanisms by which A1R mediates the effects of SD on cognitive functions and emotional regulation, potentially leading to the identification of novel therapeutic targets for sleep-related cognitive disorders.

This article was previously posted as a preprint in Research Square on May 12, 2023 (https://doi.org/10.21203/rs.3.rs-2889457/v1).

## Materials and methods

**Figure 1:**
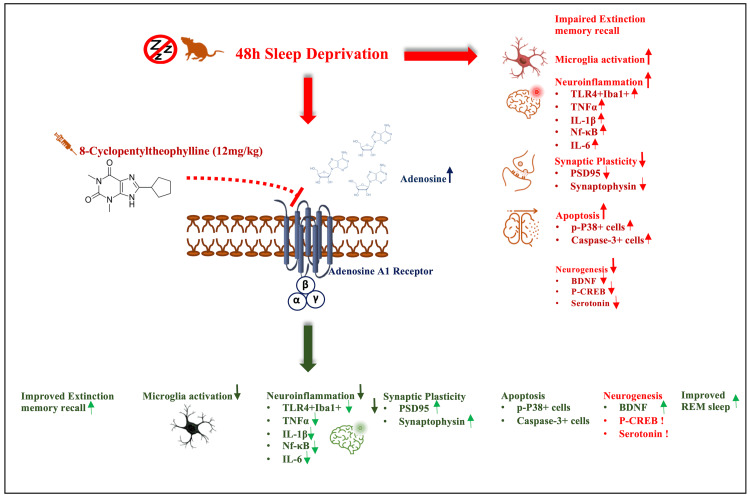
Graphical abstract of the present study

Figure 1 shows the graphical abstract of the study.

Experimental procedures

Experimental Animals

**Figure 2:**
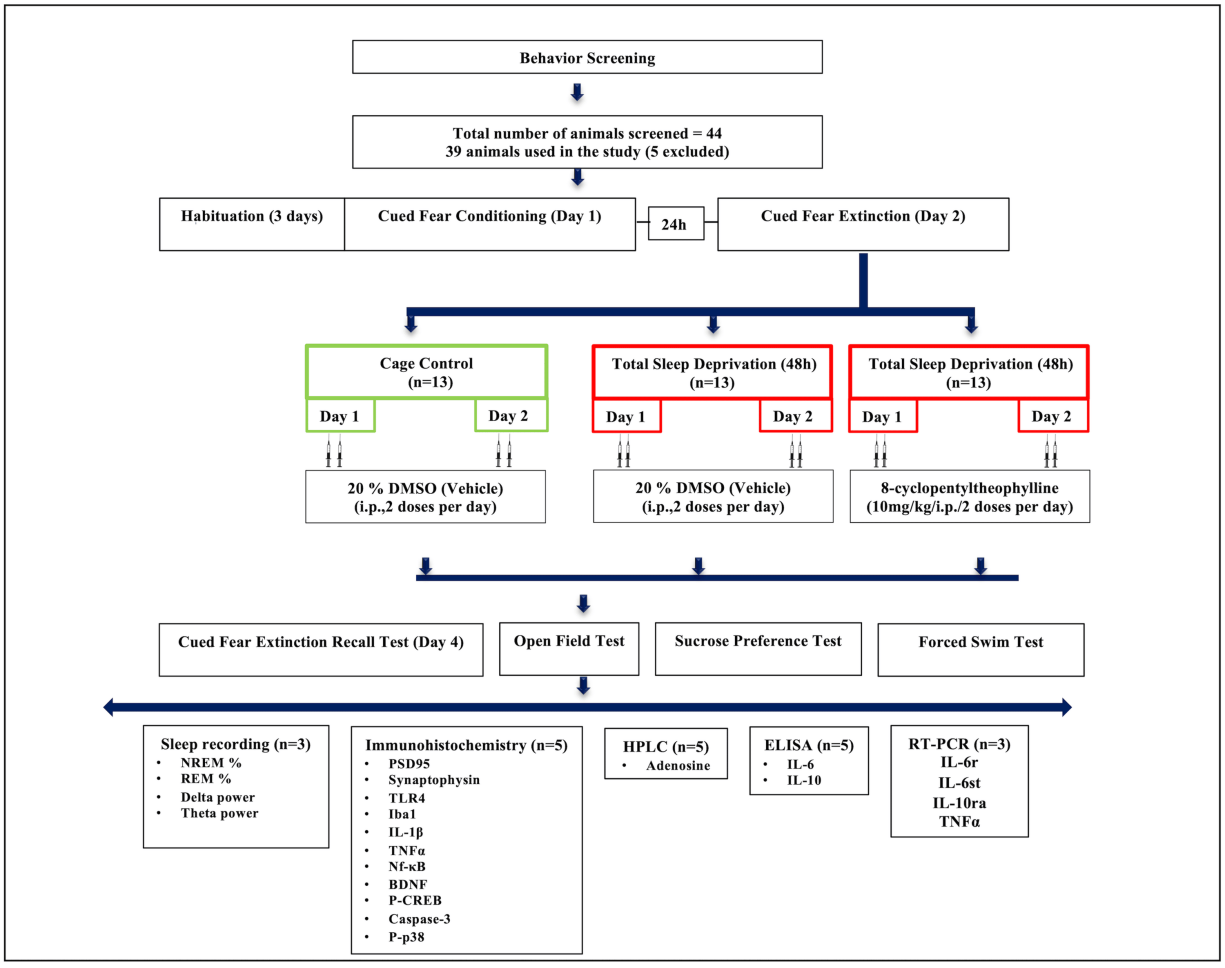
Schematic representation of the study design The study involved the following experimental timeline: 1) habituation and baseline behavioural recordings, 2) fear conditioning, 3) fear extinction training, 4) animals divided into three groups (a. CC (ad libitum sleep), b. SD (sleep deprivation for 48 hours + vehicle, i.e., 20% DMSO), c. SD + CPT (8-cyclopentyltheophylline, adenosine A1 receptor antagonist was administered for two days during 48-hour SD exposure), 5) fear extinction recall test and depressive behaviour assessment, 6) sleep architecture recording, 6) brain region dissection for molecular analyses. Out of the 44 animals screened for locomotor activity, 39 animals qualified for behavioural experiments. Five animals were excluded due to higher anxiety levels and abnormal locomotor activity. All experimental animals were handled and habituated by the experimenter, twice a day, for three days. Then, the animals were subjected to cued fear conditioning on day 1. After 24 hours, cued fear extinction training was given on day 2. Then, the animals were divided into three groups: CC (ad libitum sleep), SD (sleep deprivation for 48 hours + vehicle, i.e., 20% DMSO), SD + CPT (8-cyclopentyltheophylline, adenosine A1 receptor antagonist was administered for two days during the 48-hour SD exposure). Immediately, after the SD 48-hour exposure, all the experimental animals including control animals with ad libitum sleep were tested for the cued fear extinction recall test, open field test (OFT) on day 4, sucrose preference test for eight hours, forced swim test (FST) on day 5, followed by the dissection and collection of the brain sample. Brain samples were processed further for Immunohistochemistry (IHC), RT-PCR, ELISA, and high-performance liquid chromatography (HPLC) experiments. ‘n’ refers to sample size/number of animals in each group.

All experiments in this research employed adult male Sprague-Dawley rats (animal breeding facility, Defence Institute of Physiology & Allied Sciences (DIPAS), India) aged eight to 10 weeks and weighed 230-280 g. All the rats used were kept in filter top cages (four cage companions; floor area: 821 cm^2^, cat. no. SRC02, Orchid Scientific & Innovative India Pvt. Ltd., India). Optimal housing conditions (22 ± 2 °C, 54-60% humidity, 12-hour cycle of light and dark) were provided to all the animals. The experiments were performed in accordance with the guidelines of the Committee for the Purpose of Control and Supervision of Experiments on Animals (CPCSEA) of the Indian Government with due consideration to minimize suffering. The number of animals was approved by our Institutional Animal Ethics Committee (IAEC) (approval no. IAEC/DIPAS/2019-22). Food pellets (Lipton India Ltd., India) and water were provided ad libitum. The test animals were well-habituated with the experimenter prior to the behavioural paradigm and sample collection. All the behavioural experiments were carried out between 9:00 and 11:00 am. Before the behavioural evaluation, the rats were tested for two weeks for any behavioural abnormalities like low growth rate and disorientation resulting in the animals being excluded from the study before being divided into groups. The study was initiated by screening all experimental animals in the open field test (OFT), and a total of 44 animals were initially screened in this study. Following the screening, five animals were excluded due to less locomotor activity, and 39 animals were assigned to three groups: CC (n = 13), SD + vehicle (n = 13), SD with adenosine A1R antagonist (8-CPT) (n = 13) for behavioural analysis. Afterwards, all animals were tested for fear extinction memory recall after 48 hours of either ad libitum sleep or SD and humanely euthanized for brain sample collection for further analysis. A schematic Illustration of the study design is depicted in Figure 2.

Chemicals

All chemicals utilized in the experiments were purchased from Sigma-Aldrich, USA, unless otherwise stated specifically. Solvents and standards used for quantification were of HPLC grade. Primary antibodies were procured from multiple commercial suppliers as mentioned within the protocol.

Pharmacological Intervention

Adenosine A1R antagonist, 8-CPT, which crosses the blood-brain barrier and dissolves in 20% DMSO, was used. Dosage optimization of the adenosine A1R antagonist previously reported by our group [15] enabled us to choose the optimum dose (10 mg/kg, i.p.) of 8-CPT. 8-CPT was prepared in 20% DMSO and administered intraperitoneally at 20 mg/kg/day, divided into two doses (10 mg/kg each) at 10:00 AM and 6:00 PM during the 48-hour SD treatment. Simultaneously, the vehicle (20% DMSO in two doses, i.p.) was also injected into the control group and SD group daily.

Total SD

The 48-hour total SD protocol was based on a previously reported study from our group [16]. To achieve total SD, an automated procedure using tone and vibration stimuli based on animals was performed. This version of the automated apparatus comprises animal behaviour tracking software (ANY-maze, Stoelting, USA), Ami Interface (ANY-maze, Stoelting, USA), an infrared camera for continuous detection, vibrating pads, an amplifier, and Crowson TES100SG Stereo Tactile Motion System. Animals were positioned in sleep-deprivation Plexiglass cages with similar environmental conditions to that of the housing facility. Food and water were provided ad libitum. The software detects episodes of immobility and freezing shown by the animal. If the duration of immobility and freezing exceeded 30 seconds and five seconds, respectively, the software automatically triggered the vibration event of the cage and tones through the speakers in order to sleep-deprive the rat.

Assessment of Fear Extinction Memory Recall

Apparatus: The fear conditioning experiment was carried out in a chamber (Context A) constructed with Plexiglass cages and aluminium walls (Ugo Basile instruments, Italy) on day 1. The chamber contained a metal grid floor connected to a shock generator with a wall-mounted speaker to present the auditory stimuli and a dim light (2-3 lux). A computer interface program (ANY-maze, Stoelting, USA) monitored the delivery of foot shock by steel grid floor and auditory stimuli. An infrared video camera mounted above the chamber enabled video recording and observation. The chamber was housed inside a larger sound-attenuating box (Ugo Basile Instruments, Italy) with a background white noise level of 55 dB. Cages were sterilized using isopropyl alcohol after the completion of the trial of each animal. 

Auditory fear conditioning: The animals randomly assigned into three groups (n = 11 per group) were subjected to behavioural experiments. A pilot study was carried out before experiments to verify that both cage-control animals and sleep-deprived animals exhibit no difference in perception and processing of the foot shock or fear response to the tone per se. General indications of discomfort like jumping and/or vocalizations, expressed by both the control and sleep-deprived animals did not differ. Both groups exhibited the same kind of response at 0.5 mA of shock. 

For the auditory fear conditioning, the animals were subjected to three phases of training. On day 1, fear conditioning was performed by the presentation of conditioned stimulus (CS) (15 seconds, 10 KHz, 75 dB) and unconditioned stimulus (US) (two seconds, 0.5 mA). Rats were habituated to handling and the conditioning context for 15 minutes twice every day for three days before training on day 1. On day 1, they were trained with a single conditioning trial containing 10 presentations of a tone CS (15 seconds, 10 KHz, 75 dB) that co-terminated with a foot-shock US (two seconds, 0.5 mA). The inter-trial interval was 120 seconds. In total, 11 animals were used for each experimental group. 

Cued fear extinction: After 24 hours, on day 2, initial cue recall and subsequent cue extinction were measured in context B. Cue extinction consisted of prolonged fear extinction training, with a single trial containing 30 presentations of only CS (15 seconds, 10 KHz, 75 dB; inter-trial interval of 120 seconds). Then, the animals were subjected to three different exposure groups: CC (48-hour ad libitum sleep), SD (48-hour SD + vehicle), and SD + CPT (48-hour SD+ CPT).

Cued fear extinction recall test: On day 4, all animals were subjected to the cued fear extinction recall test and extinction recall was assessed in context B. It consisted of only three CS presentations to analyse the cued fear extinction memory recall by the amount of freezing in rats. Freezing behaviour was defined as the absence of all movements except respiration, lasting ≥2 seconds. Video files were recorded at 30 frames per second by the ANY-maze software. The baseline video image was captured and used as a calibration sample before the placement of the rats in the chamber. This calibration represents the baseline noise in the video signal on a per-pixel basis over successive video frames. After the placement of the animal in the chamber, video frames were compared with each other and with the calibration sample to detect any difference in pixels. A larger difference in pixels than the pixels in the calibration sample was recorded as animal movement. Freezing scores were generated by the ANY-maze software based on the threshold set manually and calibrated according to the freezing detection sensitivity [17]. Freezing scores were analysed by the co-authors (H.P. and G.C.) blinded to the experimental groups.

Assessment of Anxious-Depressive-Like Behaviour

Open field test (OFT): The OFT is a well-established method for evaluating anxiety-related behaviours, locomotor activity, and exploratory tendencies in rodents. The testing apparatus consists of a 55 cm x 55 cm area enclosed by walls 50 cm in height, with the floor marked by a grid of squares. During the test, rats are introduced to the corner of the apparatus and allowed up to five minutes to freely explore the area. Key parameters such as the number of line crossings, motion tracking, and instances of defecation are recorded and subsequently analysed to assess the subjects' behavioural responses

Sucrose preference test (SPT): The SPT is a widely utilized behavioural assay to investigate depression-like behaviour, capitalizing on the natural preference of rats for sweet solutions. This test was conducted following previously published protocols. Initially, rats were acclimatized with two bottles of plain water for two days, followed by a 2% sucrose solution for an additional two days in their home cage. Subsequently, the animals underwent a 24-hour deprivation period of both water and sucrose solution. On the testing day, each rat was housed individually in their home cage and provided with ad libitum access to both the 2% sucrose solution and plain water for eight hours. The absolute intake of sucrose and water was measured, and sucrose preference was calculated using the formula: preference = (sucrose intake / (water intake + sucrose intake)) × 100%.

Forced swim test (FST): The FST is a frequently utilized rodent behavioural assay to evaluate depressive-like behaviour. In this procedure, rats were placed in a transparent glass cylinder (50 cm in height and 20 cm in diameter) filled with water to a depth of 30 cm, maintained at a temperature of 23-25°C, and allowed to swim for six minutes. During the test, immobility time (periods with no noticeable movement lasting more than one second), climbing time (periods of the rats attempting to escape the cylinder), and swimming time (periods of continuous swimming using all four limbs) were recorded using the ANY-maze video tracking system.

Tissue Collection and Processing

Following the behavioural test, rats were administered an anaesthetic dose of ketamine (80 mg/kg) and xylazine (20 mg/kg). The hippocampus region was isolated from the brain, kept on ice, and rinsed with a 0.1 M phosphate-buffered saline (PBS) solution. It was then stored at -80°C. The hippocampal tissue was homogenized using a polytron homogenizer in a 1X PBS solution containing a protease inhibitor and phosphatase cocktail. The resulting mixture was centrifuged at 10,000 rpm for 15 minutes at 4°C. Carefully separating the supernatant, it was preserved at -80°C for Western blot, HPLC, and enzyme-linked immunosorbent assay (ELISA) purposes. 

Immunohistochemistry

After the 48-hour SD followed by behavioural evaluation, the brain samples were dissected from animals and were used for every immunohistochemistry (IHC) experiment. Animals from all groups were deeply anaesthetized with urethane (cat. no. U2500, Sigma-Aldrich, USA, 1.2 g/kg body weight) and perfused using chilled 0.1 M PBS followed by fixation with ice-cold 4% paraformaldehyde (PFA) (cat. no. P6148, Sigma-Aldrich, Germany). Brain tissue was isolated and processed through an increasing gradient of sucrose solution (10%, 20%, and 30%) at 4°C until it sank down completely, according to the previously described protocol [18,19]. Brain sections from all groups (CC, SD, and SD+CPT) were processed simultaneously to maximize the accuracy of comparisons across groups. Coronal brain sections were sliced using a cryostat (Leica Biosystems) and deep-frozen with OCT medium (Leica 7 Biosystems). Sections were collected in 0.1 M PBS with 0.02% sodium azide. IHC experiments were performed in 30-μm, free-floating, coronal sections for all the antibodies. Coronal brain sections of 30 μm from the dorsal hippocampus (bregma−3.14 to−4.30) were used for all staining protocols. Each section was washed with PBST (PBS with 0.1% Tween-20). Heating of the section was done for 10 minutes to retrieve the epitope in a sodium citrate solution at pH 6.0. Blocking was done for two hours in a blocking buffer comprising 0.03% Triton X-100 and 10% goat serum in PBS. We incubated the sections in appropriate primary antibodies for 24-48 hours at 4°C, followed by nuclear staining by DAPI incubation for 10 minutes. Then, sections were incubated in fluorophore-conjugated secondary antibodies (Alexa Fluor 488 and Alexa Fluor 594, Invitrogen, USA) for 2 hours at room temperature (RT). Sections were mounted on gelatin-coated glass slides for microscopy. Details of all the primary and secondary antibodies are mentioned in Table 1. Although manufacturers provided antibody validation reports, we included positive expression (high expression of the target protein) and negative control (lack of expression of the target protein) tissue sections for validating antibodies for their specificity and sensitivity towards the target antigen. All IHC images were analysed by the experimenters (H.P. and G.C.) blinded to the experimental groups. 

**Table 1:** Details of all primary and secondary antibodies: provider information and incubation protocol IHC: immunohistochemistry, RT: room temperature

Antibody name	Host	Catalog number	Provider	RRID	Dilution	Incubation
Anti-adenosine A1 receptor	Rabbit (Rb)	ab82477	Abcam	AB_2049141	1:200 (IHC)	48 h, 4°C
Anti-BDNF	Rabbit (Rb)	ab108319	Abcam	AB_10862052	1:250 (IHC)	48 h, 4°C
Anti-caspase-3	Rabbit (Rb)	PA5-77887	Invitrogen		1:500 (IHC)	48 h, 4°C
Anti-IL-1β	Rabbit (Rb)	ab9722	Abcam	AB_308765	1:500 (IHC)	48 h, 4°C
Anti-Iba1	Rabbit (Rb)	ab178846	Abcam	AB_2636859	1:500 (IHC)	48 h, 4°C
Anti-pCREB	Rabbit (Rb)	MA5-11192	Invitrogen		1:200 (IHC)	48 h, 4°C
Anti-pNF-κB	Rabbit (Rb)	ab28849	Abcam	AB_881293	1:500 (IHC)	48 h, 4°C
Anti-pP38	Rabbit (Rb)	ab4822	Abcam	AB_304659	1:200 (IHC)	48 h, 4°C
Anti-PSD-95	Rabbit (Rb)	PA5-85749	Invitrogen		1:500 (IHC)	48 h, 4°C
Anti-serotonin	Goat (Gt)	ab66047	Abcam	AB_1142794	1:500 (IHC)	48 h, 4°C
Anti-TLR4	Mouse (Ms)	ab22048	Abcam	AB_446735	1:200 (IHC)	48 h, 4°C
Anti-TNFα	Rabbit (Rb)	ab6671	Abcam	AB_305641	1:250 (IHC)	48 h, 4°C
Secondary antibodies						
Alexa Fluor 594	Rabbit (Rb)	A11037	Invitrogen		1:2000 (IHC)	2 h, RT
Alexa Fluor 488	Rabbit (Rb)	ab150077	Abcam	AB_2630356	1:2000 (IHC)	2 h, RT
Alexa Fluor 594	Mouse (Ms)	A32740	Invitrogen		1:2000 (IHC)	2 h, RT
Alexa Fluor 488	Mouse (Ms)	A32723	Invitrogen		1:2000 (IHC)	2 h, RT

Morphologic Analyses of Glial Cells (Sholl Analysis)

Microglial morphology was analysed using a previously published protocol [20]. Microglial cells in the DG, CA1, and CA3 regions were selected (six to eight cells/section; n = 6) on 30-μm hippocampal tissue sections. Concentric circles were drawn using the ImageJ (NIH, USA) concentric circle plugin. Circles were centred on the soma by manually determining the radii, which were then increased by 2 μm with every circle. Sholl analysis was performed using the ImageJ Sholl analysis plugin. These data were quantitated using the following parameters: Nm = process maximum (the maximum number of intersections for the cell) and Np = number of primary branches (the number of branches originating from the microglial soma). The ramification index (Nm/Np) was calculated to quantify the cell branching density. The cell soma area was also calculated using the ImageJ software. All surface and filament parameters were exported into separate Excel sheets and used for data analysis. 

Fluorescent Microscopy and Quantification

All images were acquired with an Upright Fluorescent microscope from Olympus (model no. BX51TF, Tokyo, Japan) using 20x and 40x objectives using the CellSens software from Olympus, Japan, and filter specifics (DAPI, FITC, Cy5, TRITC) were tailored accordingly. Quantitative analysis of the overall expression of a specific protein was done by measuring the fluorescence mean intensity per frame (Integrated Density, IntDen), which includes all three layers (molecular, granular, and sub-granular layers) of sub-regions DG, CA1, and CA3 in the ImageJ software (https://imagej.nih.gov/ij, National Institutes of Health, Bethesda, MD, USA). A total of 30 μm sections from the dorsal hippocampus were used for Iba-1, P-p38, and caspase-3 positive immune cells counting as described in the published protocols before [15,16]. The mean intensity values of the target proteins in both the left and right hippocampi were considered for analysis. To quantify the co-labelled cells, every sixth coronal section of the serially sectioned brain was immunostained (150 µm apart) from all groups. A range of 8-10 of such sections from five animals were considered for statistical comparison. Moreover, co-labelled cells in the DG, CA1, and CA3 of the dorsal hippocampus were counted manually. In ImageJ, the mean pixel intensity values for any colour vary from 0 to 255, in which 0 represents the darkest shade and 255 represents the lightest shade of the colour intensity. According to this range, mean intensity values of 0 to 60 are considered strong, from 61 to 120 is moderate, weak for 121 to 180 and negative for values higher than 180. Inter-observer variability and bias are reduced by independent analyses of image intensities by authors and discussed for agreement and discrepancies. 

High-Performance Liquid Chromatography (HPLC)

The adenosine levels in the hippocampus tissue were measured using ion-pairing reversed-phase HPLC-PDA. After 48-hour SD, brain tissue samples were sonicated in one volume (w/v) of 10% cold 0.1 M H_2_SO_4_. Two volumes of ice-cold 0.1 M Tris buffer solution and 25 μl of a 1 N NaOH solution were added and centrifuged at 15000×g at 4°C for 15 minutes. The supernatant was filtered using a syringe filter (0.22 μm, Merck Millipore, USA) and stored at -80°C for future use. Samples were then injected into the HPLC system consisting of a C-18 column (XBridge Shield RP18 5 μm; 4.6 × 250 mm) with photodiode array detection (Waters PDA 2998) ranging from 210 nm to 400 nm. The column temperature was maintained at 30°C with a run time of 10 minutes per sample at a flow rate of 0.8 ml/min in the isocratic mode. The column pressure was maintained at 1800 psi. The samples and standards were run (injection volume of 20 μl) using the autosampler (Waters 2707). The mobile phase consisted of vacuum-degassed water, methanol, and acetonitrile at 88%, 5%, and 7%, respectively, using a 0.22 μm membrane in a vacuum pump (Millipore, USA), and a degasser unit removed dissolved gases from samples and the mobile phase before injection. The adenosine peaks were detected by the photodiode array detector. The retention time was recorded and matched with that of the standards, and concentrations were estimated by extrapolating peak area values with the calibration curve (peak area vs. concentrations). 

*Real-Time Polymerase Chain Reaction* *(RT-PCR)*

The total RNA extraction from the hippocampal tissue was performed utilizing a TRIZOL reagent (Sigma, USA). The concentration and purity of the obtained RNA were assessed through a Nanodrop spectrophotometer (Thermo Fisher Scientific, USA), measuring absorbance at 260 and 280 nm. To ensure the quality and size distribution of the isolated RNA, denaturing agarose gel electrophoresis with ethidium bromide staining was conducted using a 1% agarose gel prepared in a TBE (tris-borate EDTA) buffer. Subsequently, the RNA was subjected to reverse transcription to generate complementary DNA (cDNA) employing the RT2 first strand cDNA Synthesis Kit (QIAGEN Sciences, Maryland, USA) following the manufacturer’s instructions. The ensuing cDNA served as the template for relative quantitative analysis of interleukins and tumour necrosis factor gene expression within each experimental group. This analysis was executed using the RT2 Profiler inflammatory cytokines and receptor array (QIAGEN Sciences, Maryland, USA) along with the RT2 SYBR® Green qPCR master mix (QIAGEN Sciences, Maryland, USA). Normalization of each gene's threshold cycle (Ct) values to the housekeeping genes was performed as a prerequisite for accurate comparative gene expression analysis. The final quantification and interpretation of the gene expression data were carried out utilizing dedicated software accessible online at www.sabiosciences.com

Estimation of Cytokine Levels in the Hippocampal Tissue

The concentrations of pro-inflammatory cytokines IL-6 and anti-inflammatory cytokine IL-10 in hippocampal lysates were determined by employing their corresponding ELISA kits procured from Lifespan Biosciences, Inc. (Seattle, WA, USA) and RayBiotech Life, Inc. (Georgia, USA). The assay was conducted following the prescribed protocol provided by the manufacturers.

EEG Recording 

EEG electrode implantation: Nine male Sprague-Dawley rats (250 ± 20 g; Animal Care Facility, DIPAS, Delhi) were implanted with chronic recording devices for continuous recordings of electroencephalography (EEG), electromyography (EMG), core body temperature (Tb), and locomotor activity (LMA) through telemetry. Animals were anaesthetized with ketamine (80 mg/kg body weight; Themis Medicare Ltd., India) and xylazine (10 mg/kg body weight; India Immunologicals Ltd., India), and the fur was shaved from the head and the midabdominal region. Then, the animal was placed in a stereotaxic frame (Stoelting Co, IL, USA). Core body temperature was monitored and maintained between 36.5°C and 37.5°C throughout the surgery. After disinfection of the skin using betadine and alcohol, a dorsal midline incision on the top of the head and a mid-ventral incision through the peritoneum were made along the linea alba. Sterile miniature transmitters (4-ET, Data Sciences Inc., MN, USA) were inserted through this incision closer to the ventral abdominal region and sewn to the musculature with a single stitch of sterile silk suture (4-0) to inhibit later movement inside the body. Four bio-potential leads were inserted subcutaneously into the neck and head region. Then, the abdominal musculature was sealed with an absorbable suture (Vicryl 3-0), and the peritoneum was closed with a silk suture (3-0). After the skull was completely cleaned, holes were drilled through the skull at 3.0 mm anterior to Bregma and 1.5 mm lateral to the midline [17]. The two bio-potential leads that were used as EEG electrodes were inserted into the holes and affixed to the skull with dental acrylic. The two bio-potential leads that were used as EMG electrodes were sutured into the neck musculature, and the incision was closed with a suture (silk 4-0). Surgical pain was relieved postoperatively by intramuscular injection of diclofenac sodium (15 mg/kg body weight) and intramuscular injection of ciprofloxacin hydrochloride (25 mg/kg body weight) as an antibiotic for three days. Animals were allowed to recover in clean cages and monitored until they were ambulatory. 

EEG recording and power spectral analysis: The EEG and EMG recordings were carried out continuously from 08:00 am for 48 hours in a temperature-controlled room with the customized automated SD apparatus in the laboratory. A DSI amplifier was used to amplify and filter the EEG signal at 0.1 and 30 Hz. The SD cages were positioned above the receiver plates (RPC-2, DSI, Minnesota, USA). Using Ponemah software, the amplified and filtered EEG was digitized and recorded at a sampling rate of 500 Hz. Trained scorers (B.T., K.R., and K.K.) blinded to experimental treatment conditions identified each five-second epoch as either quiet wake, active wake, NREM sleep, or REM sleep after data collection by visually analysing the EEG and EMG recordings. The EEG power spectral analysis was conducted using fast Fourier transform (FFT) in the Neuroscore software. For EEG frequencies ranging from 0.5 to 25.0 Hz, FFT analysis was emphasized to determine the EEG power (μV2) of theta (4-8 Hz) and delta (0.5-4 Hz) frequency bands. Results were averaged across five-second epochs in 0.5 Hz increments. 

Statistical Analysis

After analysing the data obtained from our pilot study, we determined the required sample size using G*Power version 3.1.9.6 (Germany). To detect differences in the fear extinction recall and neuroinflammation experiments among the groups, with a two-tailed α error of 5%, β error of 20%, and an effect size of 0.8, a minimum of five animals per group was determined to be necessary. However, two animals per group were included additionally to account for attrition and variability in response among the animals. Prior to conducting the behavioural experiments, animals were allocated randomly into three distinct experimental groups employing a random number generator function within Microsoft Excel (Microsoft Corporation, USA). After the behavioural tests, the brain tissue was subjected to molecular studies. All behavioural assessments and histological analyses were performed by authors blinded to the treatment groups to reduce observer bias. 

The statistical analyses were conducted using Prism software (GraphPad, San Diego, CA, USA). To ensure comprehensive insights into the data distribution, all numerical values were expressed as the means accompanied by their respective standard deviations (SDs). Assessment of normality was undertaken employing the Shapiro-Wilk, D'Agostino and Pearson, and Kolmogorov-Smirnov tests. In addition, quantile-quantile plots were constructed, further aiding in the visual evaluation of normality. For datasets exhibiting a departure from the Gaussian distribution, the Kruskal-Wallis test was applied. The determination of homoscedasticity and heteroscedasticity was executed employing Bartlett's and Brown-Forsythe tests. Data characterized by heteroscedasticity were aptly analysed utilizing the robust Welch analysis of variance (ANOVA). In instances of two-group comparisons, an unpaired two-tailed Student’s t-test was employed. Moreover, fear extinction recall data underwent a comprehensive two-way ANOVA, followed by Bonferroni post-hoc tests. Concomitantly, ordinary one-way ANOVA was effectively applied in tandem with Tukey’s test for multiple comparisons amongst the multiple groups. The outcomes of the statistical tests conducted are astutely elucidated within the corresponding figure legends. It is noteworthy to emphasize that all statistical analyses were unequivocally deemed statistically reliable at the threshold of a p-value less than 0.05.

## Results

A1R antagonism differentially regulates extinction recall and anxious-depressive-like states in rats

**Figure 3:**
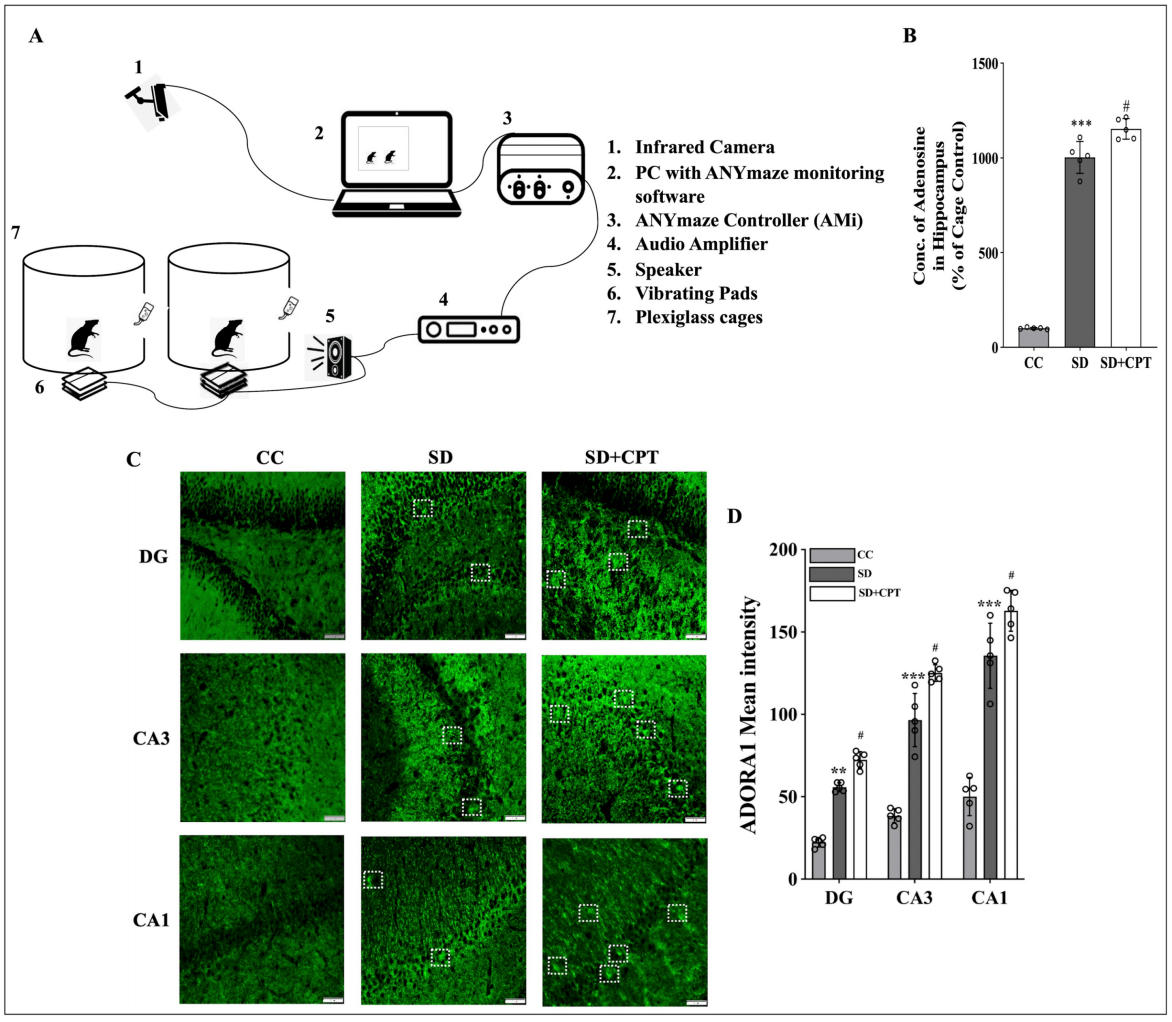
8-cyclopentyltheophylline (8-CPT) administration during 48-hour sleep deprivation increases adenosine levels and adenosine A1 receptor (A1R) expression in the hippocampus A. Schematic representation of the automated sleep deprivation (SD) apparatus for rats. B. Levels of adenosine in the hippocampal lysate measured by high-performance liquid chromatography (HPLC) demonstrating the increased accumulation of adenosine post 48-hour SD in comparison to control (F(2, 90) = 21.43; p < 0.001, n = 5). *p < 0.05 represents the comparison between CC and SD; #p < 0.05 represents the comparison between SD and SD+CPT. C. Representative IHC images. D. Statistical comparison graphs (right) of A1R expression in the DG (F(2, 9) = 164, p < 0.001, n = 5), CA3 (F(2, 9) = 130.3, p < 0.001, n = 5), and CA1 (F(2, 9) = 109.2, p < 0.001) regions. (Mean intensity by ImageJ, white scale bar = 50 μm) (one-way ANOVA, Tukey’s post-hoc test, n = 5). *p < 0.05 represents the comparison between CC and SD; #p < 0.05 represents the comparison between SD and SD + CPT; CC: cage control; SD: 48-hour sleep deprivation; SD + CPT: 48-hour sleep deprivation + 8-cyclopentyltheophylline

It has been established that SD followed by memory training dysregulates memory consolidation. Previous reports from our lab showed that 48 hours of total SD deteriorates adult neurogenesis and upsurges neuroinflammation. In continuation, to test whether adenosine A1R antagonism ameliorates SD-induced deficits in extinction memory recall, anxiety-like behaviour by OFT and depressive-like behaviours tested by SPT and FST, we sleep-deprived male Sprague-Dawley rats using our automated rodent SD system [16] (Figure 3A) and administered 8-CPT (i.p.), an adenosine A1R antagonist during SD. We wanted to estimate the levels of adenosine, as adenosine levels in the dorsal hippocampal tissue act as a prominent sleep pressure biomarker. Adenosine levels in the hippocampal lysate were quantified using HPLC and were found to be increased in SD comparison to the cage control group. In addition, adenosine A1R antagonism increases adenosine levels as compared to SD (p < 0.05) and control rats (F(2, 9) = 21.43; p < 0.001, n = 5; Figure 3B). Consequently, we analysed the expression of A1R in the hippocampal niche. We observed that A1R expression significantly increased in the DG (F(2, 9) = 164, p < 0.001, n = 5; Figure 3D), CA3 (F(2, 9) = 130.3, p < 0.001, n = 5; Figure 3D) and CA1 (F(2, 9) = 109.2, p < 0.001, n = 5; Figure 3D) of the SD and SD + CPT rats as compared to the control animals.

**Figure 4:**
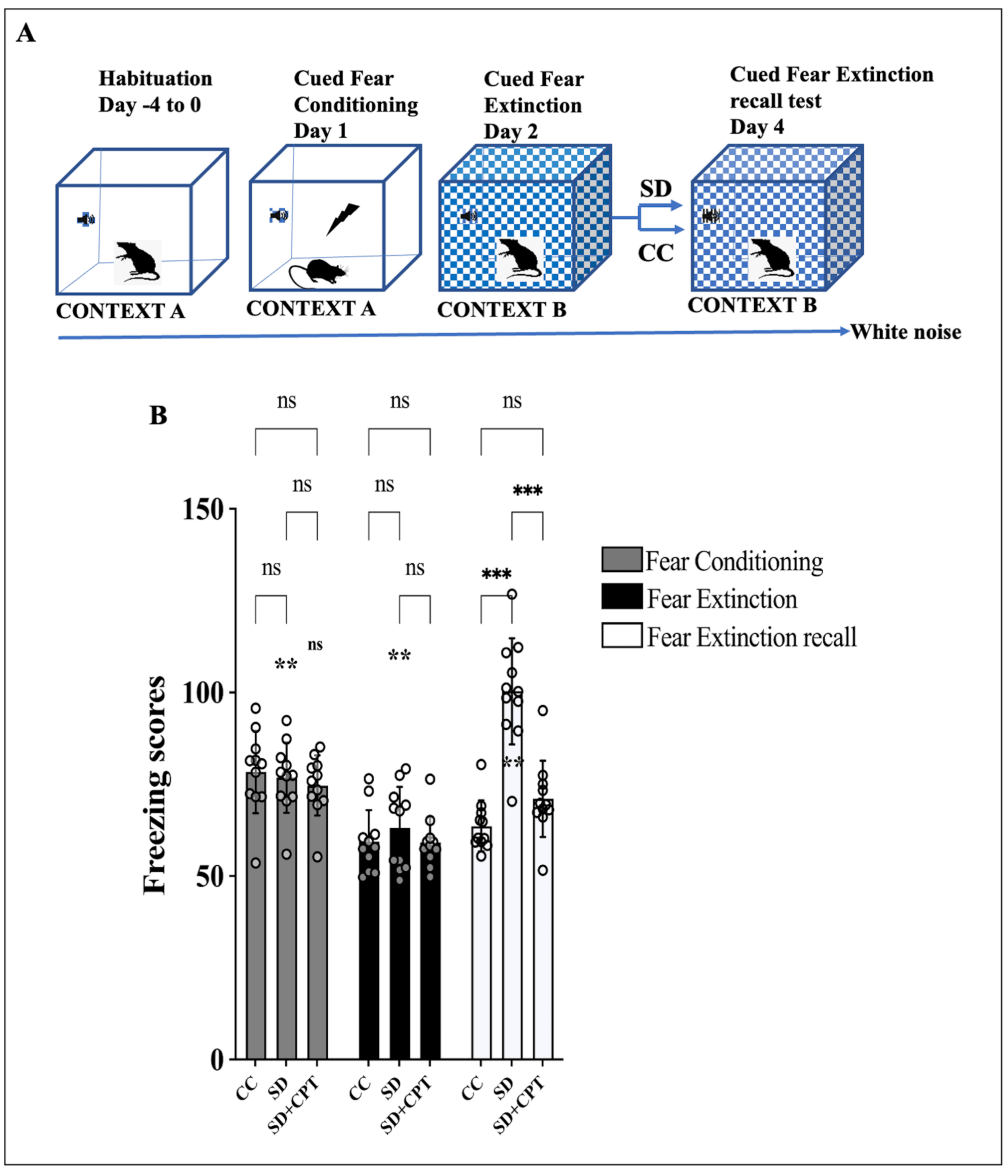
Adenosine adenosine A1 receptor (A1R) antagonism rescues sleep deprivation (SD)-induced fear extinction memory recall deficits A. Schematic representation of fear conditioning and cued extinction recall paradigm. B. Statistical comparison graphs (two-way ANOVA, Tukey’s post-hoc, n = 11) depicting the freezing scores exhibited during fear conditioning (day 1), fear extinction (day 2), and fear extinction recall (day 4) post either 48-hour sleep deprivation with vehicle (DMSO) (F(4, 60) = 15.8; p < 0.001; n = 11) or A1R antagonist CPT (p = 0.01; n = 11), and ad libitum sleep (cage control group). *p < 0.05 represents the comparison between CC and SD; #p < 0.05 represents the comparison between SD and SD + CPT. CC: cage control; SD: 48-hour sleep deprivation; SD + CPT: 48-hour sleep deprivation + 8-cyclopentyltheophylline

**Figure 5:**
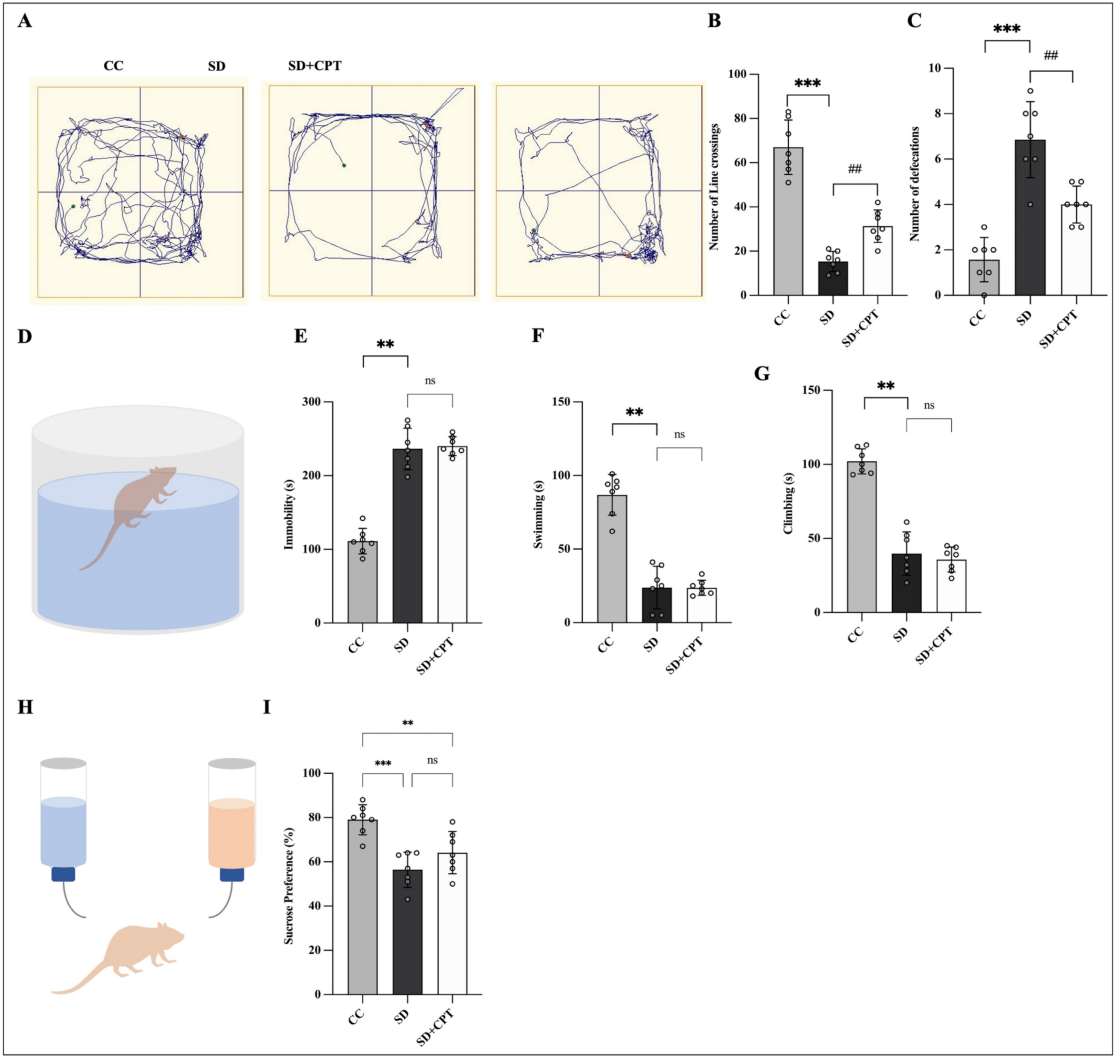
8-cyclopentyltheophylline (8-CPT) shows no effect on anxious-depressive-like behaviours in sleep-deprived rats A. Track plot in open field test (OFT). B-C. Statistical comparison graphs (bottom) (one-way ANOVA, Tukey’s post-hoc, n = 7) depicting the number of line crossings and number of defecations in exhibited during OFT. D. Schematic representation of the forced swim test. Statistical comparison graphs (bottom) (one-way ANOVA, Tukey’s post-hoc, n = 7) depicting E. time spent in immobility, F. swimming, and G. climbing in seconds. H. Schematic representation of the sucrose preference test I. Statistical comparison graphs (bottom) (one-way ANOVA, Tukey’s post-hoc, n = 7) depicting the percent of sucrose preference for 24 hours. *p < 0.05 represents the comparison between CC and SD; #p < 0.05 represents the comparison between SD and SD + CPT. CC: cage control; SD: 48-hour sleep deprivation; SD + CPT: 48-hour sleep deprivation + 8-cyclopentyltheophylline

For the assessment of fear extinction memory recall, animals were divided into three groups and habituated to the cued fear conditioning chamber and were blinded to the experimenter. We submitted animals to cued fear conditioning (context A) and cued fear extinction (context B) on day 1 and day 2, respectively, after cued fear extinction training on day 2. After 48 hours of SD and SD with the administration of 8-CPT, on day 4, during the cued fear extinction recall test (context B) (Figure 4A), SD rats exhibited a selective increase in conditioned freezing to CS as compared to the cage-control rats (F(4, 9) = 12.74; p < 0.001, n = 11, Figure 4B). Interestingly, we observed that antagonism of A1R (SD + CPT) reverted the deficits in fear extinction memory recall reflected by the reduced freezing scores on day 4 (p = 0.01, Figure 4B). In addition, it was observed that A1R antagonism increased line crossings in the OFT (F(2, 18) = 65.3; p < 0.01; Figure 5B) and reduced defecations (F(2, 18) = 33.2; p < 0.01; Figure 5C) post SD. Next, we assessed the role of A1R in modulating depressive-like behaviour following SD and found no significant difference in immobility (p = 0.9386; Figure 5E), swimming (p = 0.9997; Figure 5F), time spent in climbing (p = 0.7596; Figure 5G), and anhedonic behaviour (p = 0.2102; Figure 5I) between SD and SD + CPT in comparison to CC. These results suggest that adenosine A1R plays a vital role in the regulation of extinction memory and not anxiety and depressive-like behaviour post-SD. 

These data suggest that SD post-extinction training dysregulates memory consolidation and induces anxiogenesis and depressive-like behaviour. However, antagonism of adenosine A1Rs during SD enhanced the consolidation of extinction memory and reduced anxiety, and no prominent difference was found in depressive-like behaviour. Furthermore, it is notable from our immunostaining results that A1R antagonism resulted in the upregulation of A1R in the hippocampal niche during SD. 

SD modulates synaptic plasticity via A1R

**Figure 6:**
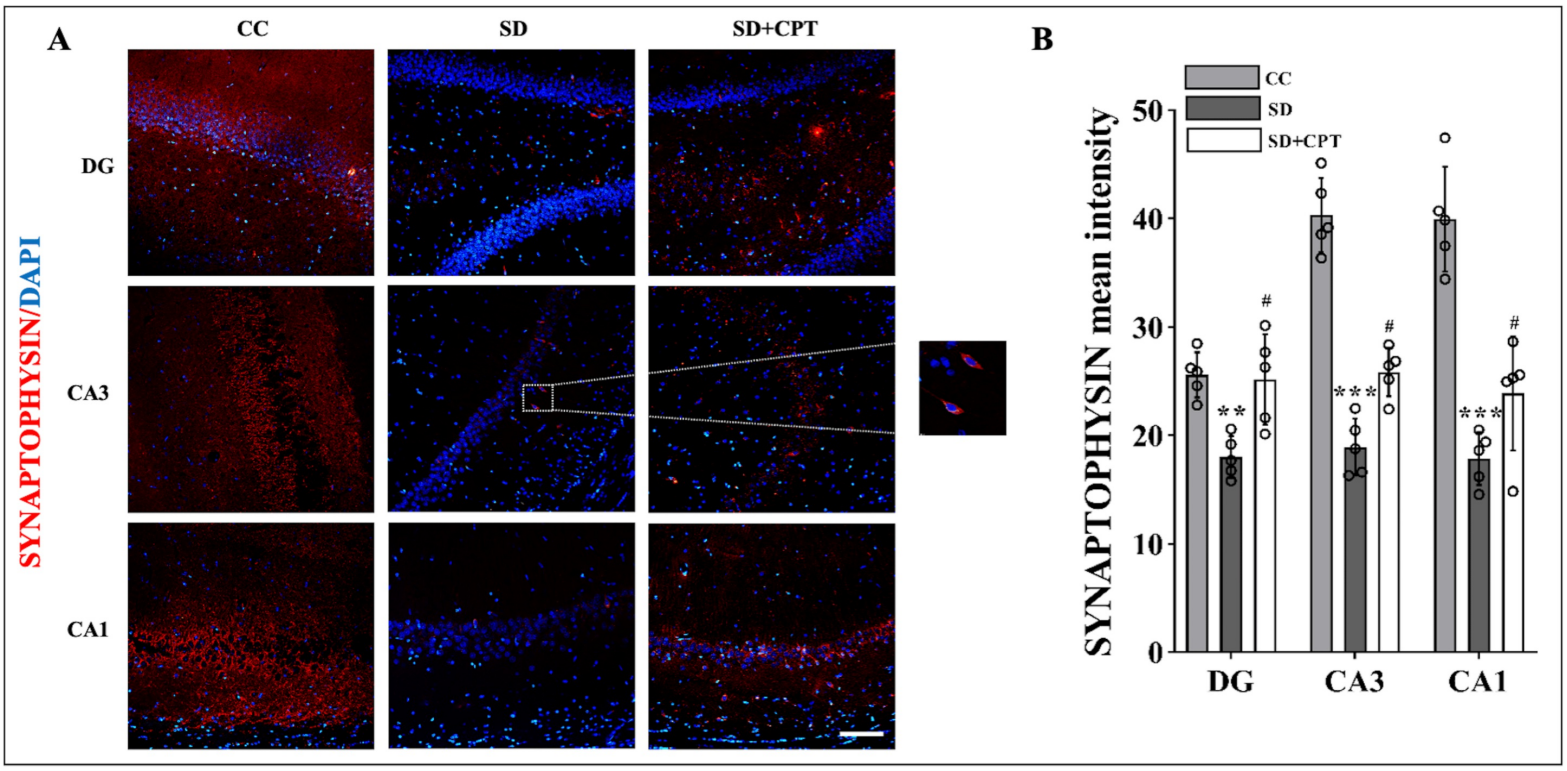
Pharmacological inhibition of adenosine A1 receptor rescues the expression of synaptophysin during 48-hour sleep deprivation (SD) A. Representative immunohistochemistry (IHC) images. B. Statistical analysis of synaptophysin expression (mean intensity by ImageJ) in the DG (F(2, 9) = 3.618; p < 0.001), CA3 (F(2, 9) = 0.6134; p < 0.001), and CA1 (F(2, 9) = 0.284; p < 0.001) region after 48-hour total sleep deprivation and DG (F(2, 9) = 3.618; p = 0.02), CA3 (F(2, 9) = 0.6134; p = 0.007) in the adenosine A1 antagonist group. (Scale bar, 50 μm; one-way ANOVA, Tukey’s post-hoc test; n = 5)

**Figure 7:**
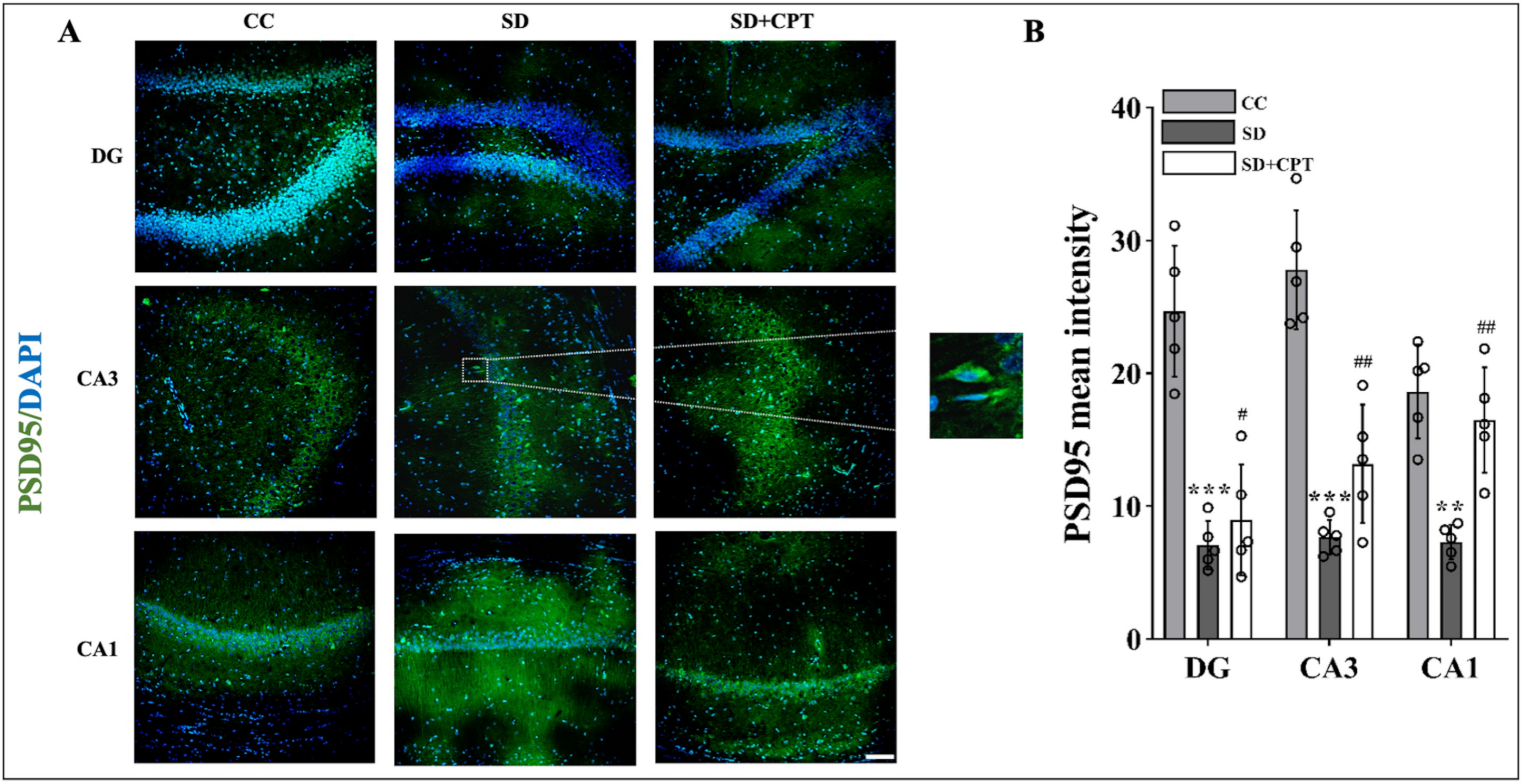
A1R prevents SD-induced downregulation of the PSD-95 expression A. Representative IHC images. B. Statistical analysis of PSD95 expression (mean intensity by ImageJ) in the DG (F(2, 9) = 2.387; p < 0.001), CA3 (F(2, 9) = 2.77; p < 0.001), and CA1 (F(2, 9) = 1.024; p < 0.001) region after 48 hours of total sleep deprivation and adenosine A1R antagonism CA1 (F(2, 9) = 1.024; p < 0.05). (white scale bar, 50 μm; one-way ANOVA, Tukey’s post-hoc test, n = 5). CC: cage control; SD: 48-hour sleep deprivation; SD + CPT: 48-hour sleep deprivation + 8-cyclopentyltheophylline

Next, we aimed to investigate whether the deficits in extinction memory recall caused by SD could be mediated by the A1 receptor. We reasoned that the altered expression of synaptic plasticity proteins like PSD95 and synaptophysin in the dorsal hippocampal niche may corroborate with impaired fear extinction recall post 48-hour SD and improved extinction recall due to 8-CPT administration. To assess this hypothesis, immunofluorescence quantification of PSD95 and synaptophysin in the dorsal hippocampal niche was carried out. We found that 48-hour SD diminished the expression of synaptophysin reduced in DG (F(2, 9) = 3.618; p < 0.001, Figure 6A, 6B), CA3 (F(2, 9) = 0.6134; p < 0.001, Figure 6A, 6B), and CA1 (F(2, 9) = 0.284; p < 0.001, Figure 6A, 6B). Furthermore, the expression of PSD95 in DG (F(2, 9) = 2.387; p < 0.001, Figure 7A, 7B), CA3 (F(2, 9) = 2.77; p < 0.001, Figure 7A, 7B), and CA1 (F(2, 9) = 1.024; p < 0.001, Figure 7A, 7B), significantly decreased in comparison to control animals. Then, adenosine A1R antagonist administration mitigated the decrement in the expression of PSD95 and synaptophysin in the hippocampal niche. Interestingly, we found that the expression of PSD95 is re-established nearly to that of control conditions only in CA1 (F(2, 9) = 1.024; p < 0.05, Figure 7A, 7B) but not CA3 and DG although the expression levels enhanced after 8-CPT administration. However, the expression of synaptophysin was elevated in both CA3 (F(2, 9) = 0.6134; p = 0.007, Figure 6A, 6B) and DG (F(2, 9) = 3.618; p = 0.02, Figure 6A, 6B) post 8-CPT administration. These results cumulatively confer that the adenosine A1R antagonism may have reversed the fear extinction memory recall deficits by re-establishing synaptic plasticity proteins in the dorsal hippocampal niche.

Adenosine A1R contributes to the regulation of neuroinflammation during SD

**Figure 8:**
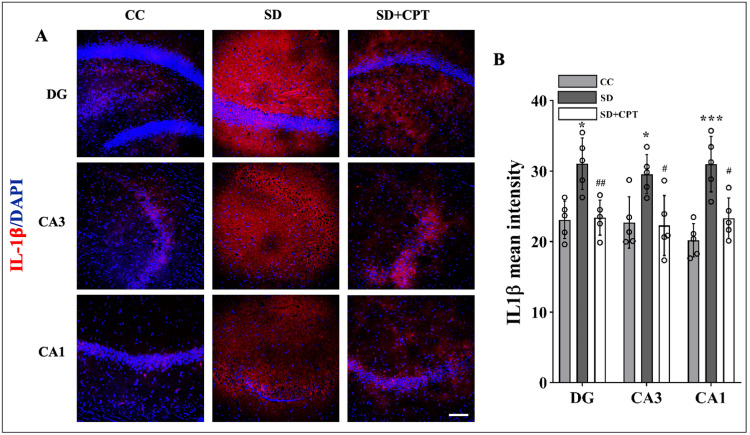
Adenosine A1 receptor (A1R) antagonism attenuates sleep deprivation (SD)-induced increase in IL-1β expression A. Representative IHC images and B. statistical analysis of IL-1β expression (mean intensity by ImageJ) in the DG (F(2, 9) = 14.79; p < 0.001), CA3 (F(2, 9) = 7.142; p < 0.01), and CA1 (F(2, 9) = 6.853; p < 0.01) regions after 48 hours of total sleep deprivation (white scale bar, 50 μm) (one-way ANOVA, Tukey’s post-hoc test, n = 5).

**Figure 9:**
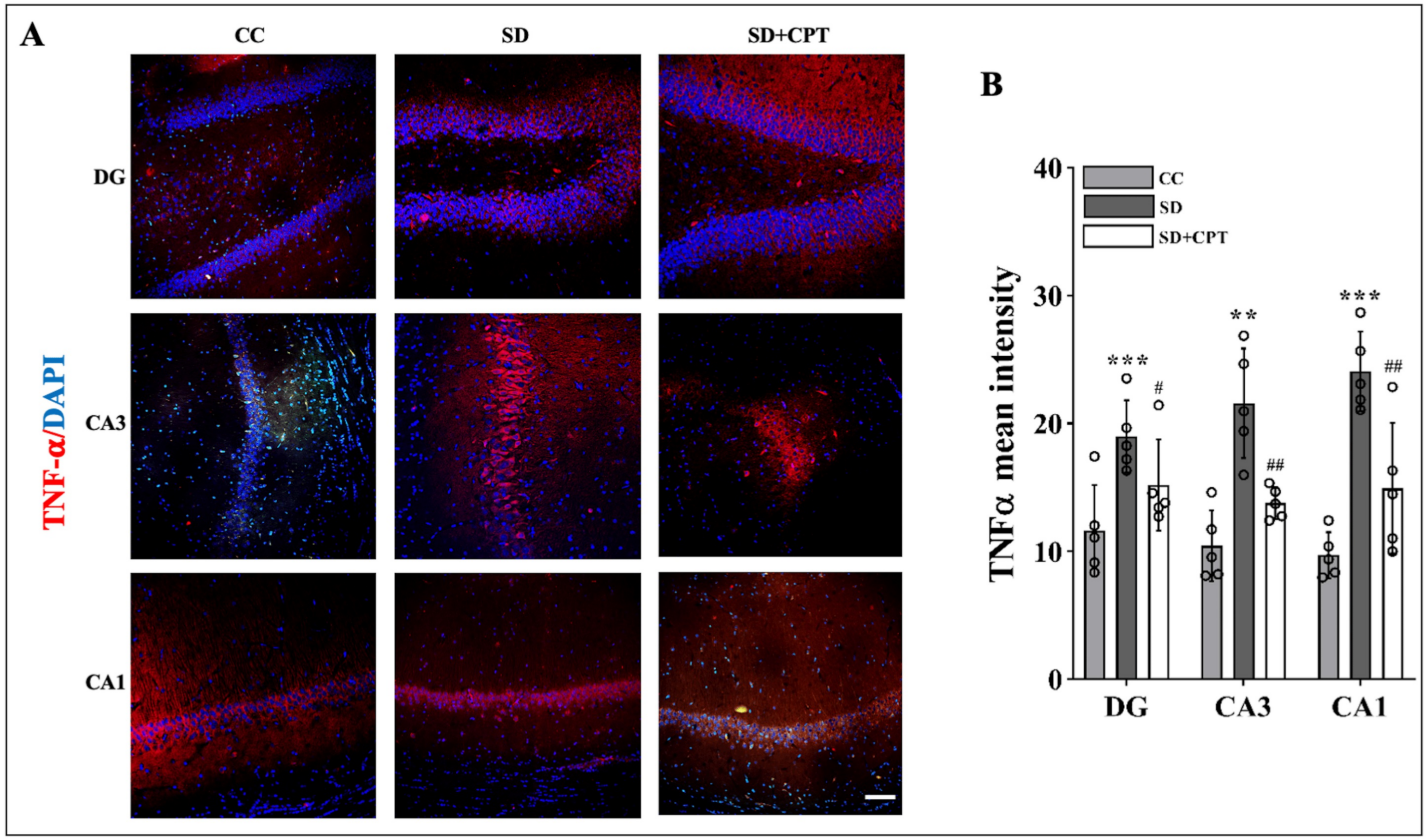
Adenosine A1 receptor (A1R) antagonism attenuates sleep deprivation (SD)-induced increase in TNFα expression A. Representative IHC images and B. statistical analysis of TNFα expression (mean intensity by ImageJ) in the DG (F(2, 9) = 32.13, p < 0.001), CA3 (F(2, 9) = 20.14, p < 0.001), and CA1 (F(2, 9) = 44.16, p < 0.01) regions after 48 hours of total sleep deprivation. (one-way ANOVA, Tukey’s post-hoc test, n = 5) (scale bar, 50 μm)

**Figure 10:**
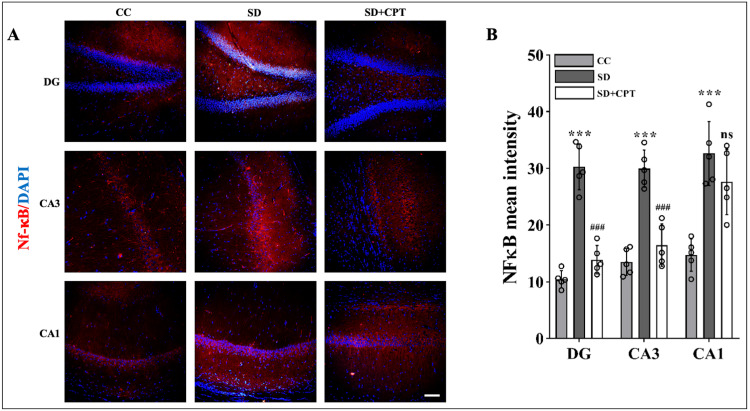
Adenosine A1 receptor (A1R) antagonism attenuates sleep deprivation (SD)-induced increase in the p-NF-ΚB S536 expression A. Representative IHC images and B. statistical analysis of p-NF-ΚB S536 expression (mean intensity by ImageJ) in the DG (F(2, 9) = 18.7, p < 0.01), CA3 (F(2, 9) = 34.13, p < 0.001), and CA1 (F(2, 9) = 142.9, p < 0.001) regions after 48 hours of total sleep deprivation. (one-way ANOVA, Tukey’s post-hoc test, n = 5) (scale bar, 50 μm). CC: cage control; SD: 48-hours sleep deprivation; SD + CPT: 48-hour sleep deprivation + 8-cyclopentyltheophylline

Previously, we demonstrated that A1R antagonism increased the expression of A1R (Figure 3D). Studies on deciphering the role of A1R showed that neuroinflammation is attenuated by the upregulation of A1R in multiple sclerosis [21]. It is well established that the hippocampal microenvironment alters during SD due to the release of inflammatory cytokines leading to neuroinflammation. However, the possibility of SD-induced neuroinflammation by the adenosine A1R remains unknown. To test the effects of A1R antagonism on SD-induced neuroinflammation, we analysed the expression of IL-1β, TNFα, and p-NFκB s536. We observed an increment in the expression of IL-1β (DG: F(2, 9) = 14.79; p < 0.001, CA3: F(2, 9) = 7.142; p < 0.01, CA1: F(2, 9) = 6.853; p < 0.01, Figure 8A, 7B; n = 5), TNFα (DG: F(2, 9) = 32.13, p < 0.001, CA3: F(2, 9) = 20.14, p < 0.001, CA1: F(2, 9) = 44.16, p < 0.01, Figure 9A, 8B, n = 5), and p-NFκB s536 (DG: F(2, 9) = 18.7, p < 0.01, CA3: F(2, 9) = 34.13, p < 0.001, CA1: F(2, 9) = 142.9, p < 0.001, Figure 10A, 9B, n = 5) in DG, CA3, and CA1 of the hippocampal niche after 48-hour SD. These findings establish the role of A1R in regulating SD-induced neuroinflammation in the hippocampus of rats. 

Differential expression of pro-inflammatory and anti-inflammatory cytokines following adenosine A1R inhibition during SD

In consideration of antecedent investigations within our laboratory, it has been discerned that pro-inflammatory and anti-inflammatory cytokines manifest disparate expression profiles during SD and subsequent minocycline administration. In this study, the role of A1R in regulating SD-induced neuroinflammation (protein level) in the hippocampus has been successfully established.

**Figure 11:**
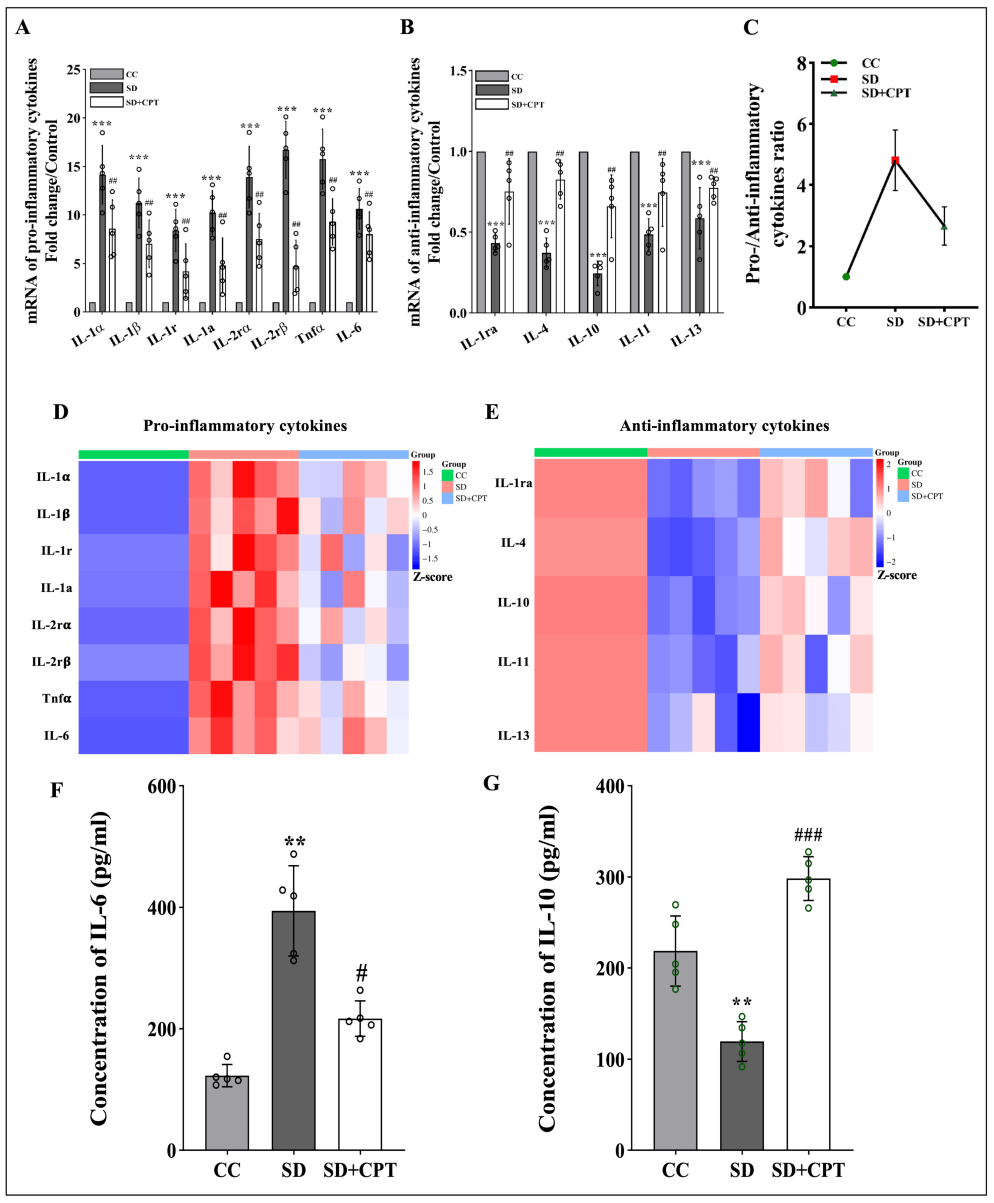
Pro- and anti-Inflammatory cytokines are differentially expressed following adenosine A1R inhibition during SD A. Statistical comparison of fold changes in the mRNA levels of pro-inflammatory cytokines in the hippocampus.*p < 0.05 when compared to control treated with vehicle, #p < 0.05 when compared to sleep-deprived treated with vehicle. B. Statistical comparison of fold changes in the mRNA levels of anti-inflammatory cytokines in the hippocampus. *p < 0.05 when compared to control treated with vehicle, #p < 0.05 when compared to sleep-deprived treated with vehicle. C. Statistical comparison of ratio of pro-/anti-inflammatory cytokines in hippocampus (n = 5) D. Heat map of differentially expressed pro-inflammatory cytokine genes. E. Heat map of differentially expressed anti-inflammatory cytokine genes. Statistical comparisons of concentrations of F. IL-6 levels (p < 0.05) and G. IL-10 levels (p < 0.05) in hippocampal lysates quantified by respective ELISA kits (one-way ANOVA, Tukey’s post-hoc test, n = 5). CC: cage control; SD: 48-hour sleep deprivation; SD + CPT: 48-hour sleep deprivation + 8-cyclopentyltheophylline

To further validate our immunostaining results, we tested the role of A1R in modulating the expression profiles of pro- and anti-inflammatory cytokine genes within the hippocampus following SD by employing real-time polymerase chain reaction (RT-PCR). The outcomes revealed a clear augmentation in the expression levels of pro-inflammatory cytokine genes including IL-1α (F(2, 12) = 36.3; ***p < 0.001; ##p < 0.01; Figure 11A), IL-1β (F(2, 12) = 31.6; ***p < 0.001; #p < 0.05; Figure 11A), IL-1r (F(2, 12) = 16.2; ***p = 0.003; #p < 0.05; Figure 11A), IL-1a (F(2, 12) = 24.7; ***p < 0.001; ##p < 0.01; Figure 11A), IL-2rα (F(2, 12) = 36; ***p < 0.001; ##p < 0.01; Figure 11A), IL-2rβ (F(2, 12) = 63; ***p < 0.001; ###p < 0.001; Figure 11A), Tnf-α (F(2, 12) = 53; ***p < 0.001; ##p < 0.01; Figure 11A), and IL-6 (F(2, 12) = 38; ***p < 0.001; ##p < 0.01; Figure 11A). Furthermore, our investigation revealed a concurrent diminution in the expression levels of anti-inflammatory cytokines during SD within the hippocampus. Notably, reduced expressions were observed in IL-1ra (F(2, 12) = 27; ***p < 0.001; ##p < 0.01; Figure 11B), IL-4 (F(2, 12) = 67; ***p < 0.001; ##p < 0.01; Figure 11B), IL-10 (F(2, 12) = 49; ***p < 0.001; ##p < 0.01; Figure 11B), IL-11 (F(2, 12) = 18; ***p < 0.001; #p < 0.05; Figure 11B) and IL-13 (F(2, 12) = 15; ***p < 0.001; Figure 11B) during SD in the hippocampus. We observed that A1R antagonism during SD led to a discernible downregulation of pro-inflammatory cytokines (Figure 11A) and a concomitant upregulation of anti-inflammatory cytokines (Figure 11B) within the hippocampus. Furthermore, an assessment of the pro- and anti-inflammatory cytokine ratio revealed a notable reduction in A1R antagonism in comparison to sleep-deprived animals (Figure 11C). To the best of our knowledge, our study is the first to directly report on the gene expression of inflammatory cytokines in the hippocampus of sleep-deprived rats administered with an A1R antagonist. Furthermore, we quantified the protein levels of IL-6 and IL-10 in the hippocampus and observed a decrease in the pro-inflammatory cytokine IL-6 (F(2, 6) = 13.87; p < 0.05, Figure 11F; n = 5) and an increase in the anti-inflammatory cytokine IL-10 (F(2, 6) = 36.37; p < 0.001, Figure 11G; n = 5) following the administration of an A1R antagonist in sleep-deprived rats.

**Figure 12:**
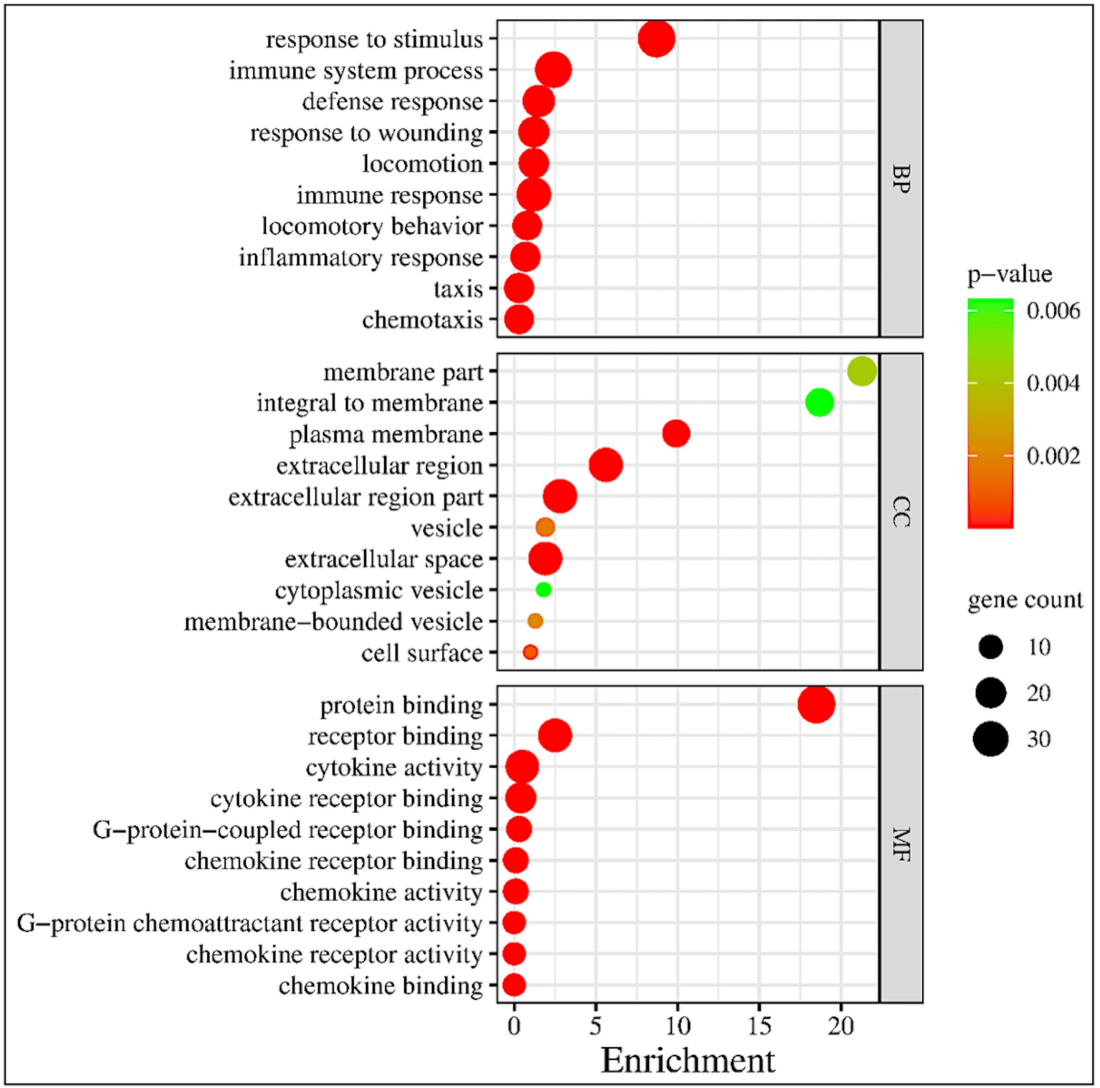
Network enrichment analysis of inflammatory cytokine genes during SD Bubble plot representing the gene ontology terms: enriched biological processes (BP), cellular component (CC), molecular function (MF), and gene counts. **p < 0.01.

Moreover, a functional analysis of differentially expressed inflammatory cytokine genes was conducted, employing a stringent threshold cut-off with a p-value of <0.01 in the Cytoscape (version 3.4.0, Seattle, USA) software. Our investigation delved into Gene Ontology (GO) enrichment terminologies, encompassing biological processes (BPs), cellular components (CCs), and molecular functions (MFs), to elucidate the functional implications of the identified genes during both SD and A1R antagonism in sleep-deprived conditions. The analysis of BPs unveiled a substantial enrichment in immune system processes (enrichment score: 2.4, p-value: 3.06E-42; Figure 12), characterized by the heightened activity in immune response pathways (enrichment score: 1.2, p-value: 4.46E-41; Figure 12). In addition, noteworthy enrichments were discerned in processes related to chemotaxis (enrichment score: 0.3, p-value: 1.33E-35; Figure 12), taxis (enrichment score: 0.3, p-value: 1.33E-35; Figure 12), and responses to various stimuli (enrichment score: 8.7, p-value: 5.46E-33; Figure 12). Collectively, these observations show a strong cellular defence and mitigation of expression of inflammatory cytokines during A1R antagonism in sleep-deprived rats. 

In the analysis of CCs, notable attention was directed towards extracellular compartments, with both extracellular space (enrichment score: 1.9, p-value: 3.86E-40; Figure 12) and extracellular region part (enrichment score: 2.8, p-value: 2.40E-37; Figure 12) exhibiting substantial enrichments. Furthermore, a significant enrichment in plasma membrane components (enrichment score: 9.9, p-value: 2.08E-05; Figure 12) underscored the integral role of membrane structures in the delineated processes. The molecular function (MF) analysis uncovered intricate interactive networks, as evidenced by pronounced enrichments in cytokine activity (enrichment score: 0.5, p-value: 4.89E-54; Figure 12), protein binding (enrichment score: 18.5, p-value: 4.09E-28; Figure 12), and receptor binding (enrichment score: 2.5, p-value: 1.51E-35; Figure 12). Collectively, these findings reveal the intricate molecular mechanisms governing the previously observed amelioration of neuroinflammation upon A1R antagonism, providing valuable insights into the regulatory networks and cellular processes.

8-CPT reduces TLR-4-mediated microglial activation during SD

**Figure 13:**
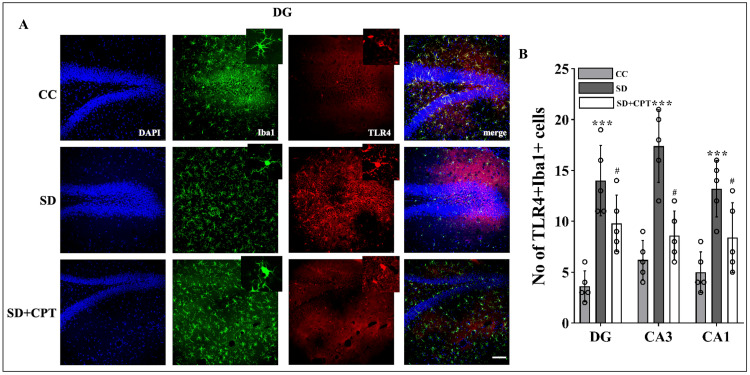
Adenosine A1 receptor (A1R) rescues 48-hour sleep deprivation (SD)-induced upsurge in TLR4-mediated microglial activation in the dentate gyrus of the hippocampus A. Co-labelling images of TLR4 (red), Iba1 (green), and TLR4+ Iba1+ (yellow) in the DG region of the CC, SD, and SD + CPT groups. B. Statistical comparison of the TLR4 expression (mean intensity in ImageJ) in the DG, CA3, and CA1 regions of the CC, SD, and SD + CPT groups (mean intensity by ImageJ). (Scale bar, 50 μm) (One-way ANOVA, Tukey’s post-hoc test, n = 5). CC: cage control; SD: 48-hour sleep deprivation; SD + CPT: 48-hour sleep deprivation + 8-cyclopentyltheophylline

**Figure 14:**
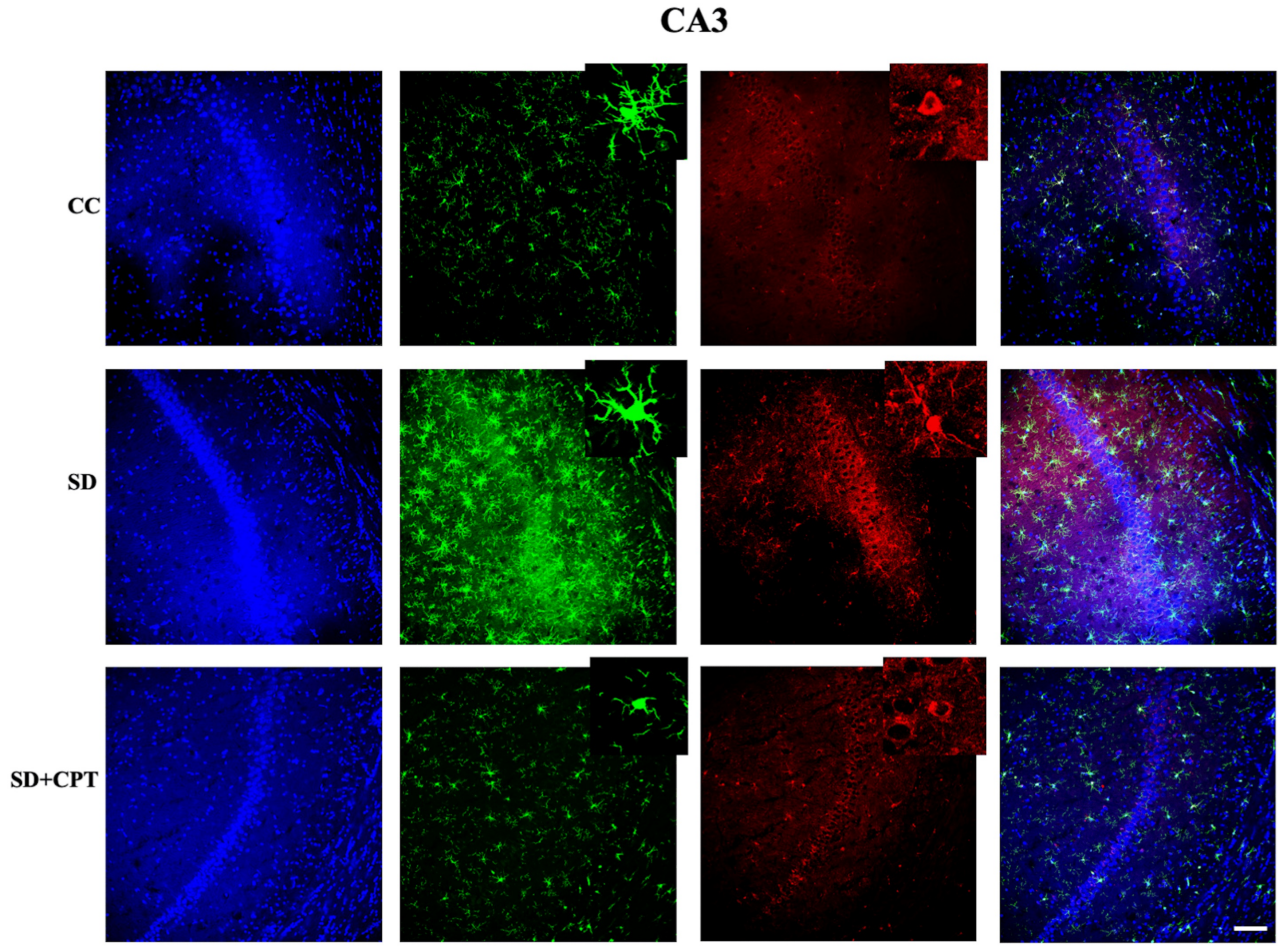
Adenosine A1 receptor (A1R) rescues 48-hour sleep deprivation (SD)-induced upsurge in TLR4-mediated microglial activation in the CA3 region of the hippocampus Co-labelling images of TLR4 (red), Iba1 (green), and TLR4 + Iba1+ (yellow) in the CA3 region of the CC, SD, and SD+CPT groups. (Mean intensity by ImageJ, scale bar, 50 μm) (one-way ANOVA, Tukey’s post-hoc test, n = 5)

**Figure 15:**
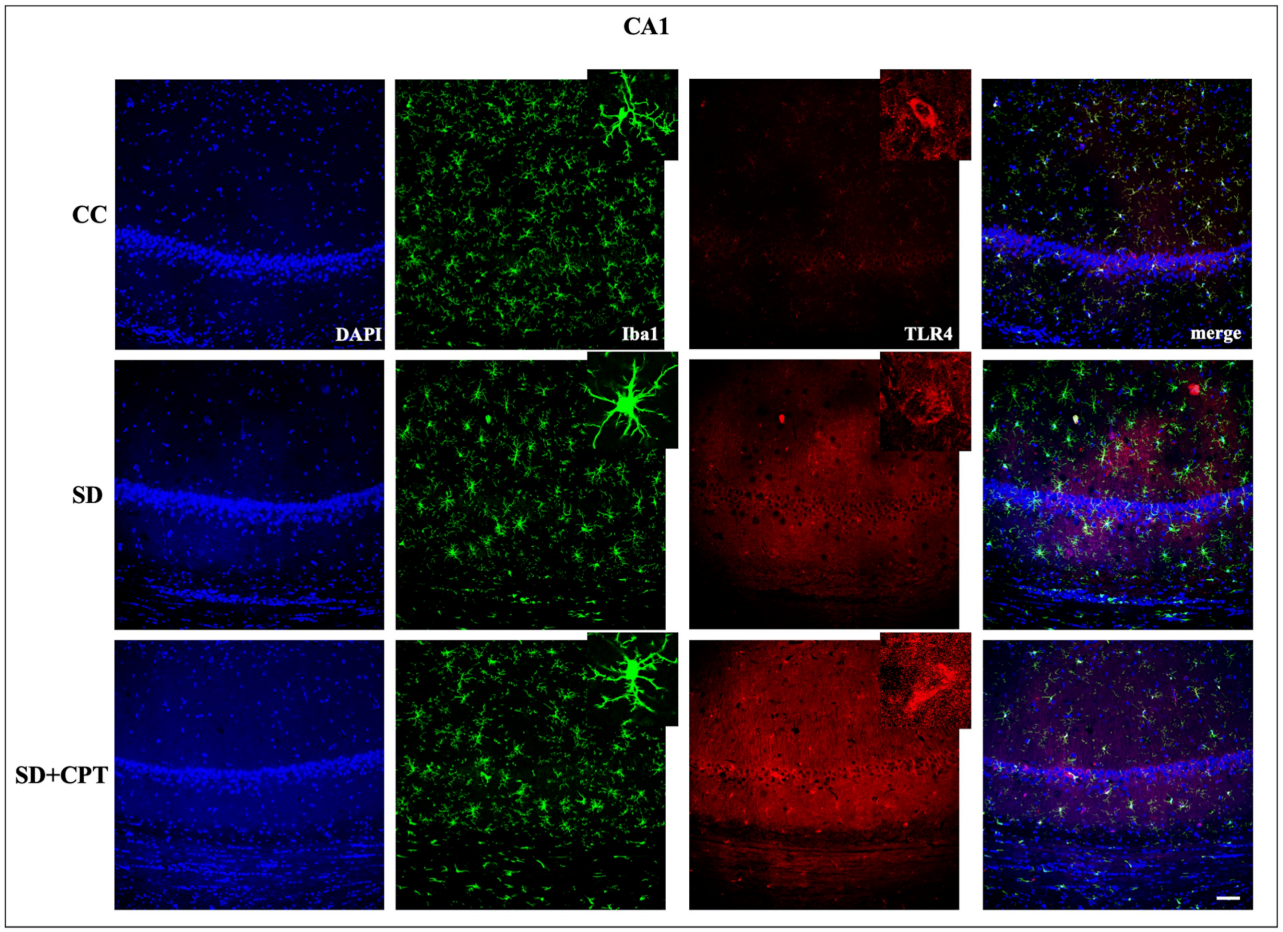
Adenosine A1 receptor (A1R) rescues 48-hour sleep deprivation (SD)-induced upsurge in TLR4-mediated microglial activation in the CA1 region of the hippocampus Co-labelling images of TLR4 (red), Iba1 (green), and TLR4 + Iba1+ (yellow) in the DG region of the CC, SD, and SD+CPT groups (scale bar, 50 μm).

**Figure 16:**
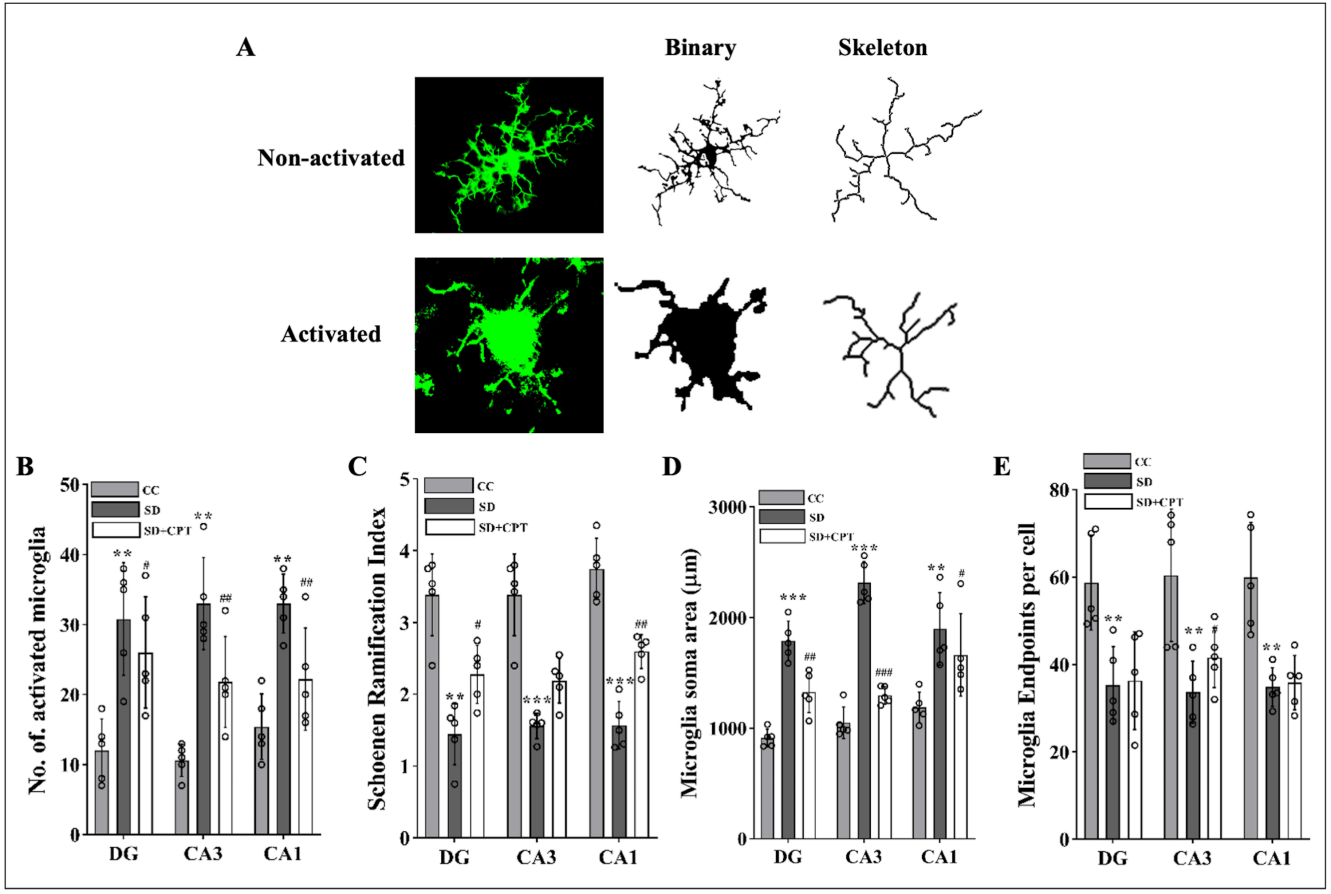
Adenosine A1 receptor (A1R) antagonism suppresses sleep deprivation (SD)-induced microglial activation A. Representative images of non-activated and activated microglia in hippocampus. B. Statistical comparison of the no. of activated microglia. C. Shoenen ramification index. D. Microglia soma area. E. Microglial endpoints per cell. *p < 0.05 denotes the comparison of the SD group with control; #p < 0.05 denotes the comparison of the SD + CPT group with the SD group treated with the vehicle. CC: cage control; SD: 48-hour sleep deprivation; SD + CPT: 48-hour sleep deprivation + 8-cyclopentyltheophylline. (One-way ANOVA, Tukey’s post-hoc test, n = 5)

It is evident from several studies that the TLR4 receptor mediates inflammatory cascades in both acute and chronic neurological stress. Next, we investigated whether A1R regulates SD-induced TLR4-mediated microglial activation. We observed that SD led to an increment in TLR4 expression in DG (F(2, 9) = 47.55; p < 0.001; Figure 13A, 13B), CA3 (F(2, 9) = 43.86; p < 0.001, Figures 13, 13B), and CA1 (F(2, 9) = 55.21; p < 0.001, Figures 15, 13B). Administration of 8-CPT reduced the TLR4 expression in the hippocampal niche (p < 0.01, Figures 13-15) of SD rats. We also observed an upsurge in the number of Iba1-positive cells in DG, CA3, and CA1 following SD, which was reverted by 8-CPT (Figures 13-15). These data intrigued us to examine the co-expression of TLR4 and Iba1 post SD. It led to an interesting observation that the number of co-labelled cells positive for TLR4 and Iba1 increased in SD animals (Figure 13B) as compared to control animals. The two-way ANOVA results showed that both the hippocampal regions (CA1, CA3, and DG) and the treatment groups (CC, SD, and SD+CPT) had significant main effects. Specifically, the hippocampal regions impacted TLR4+Iba1+ co-labelled cell expression, contributing to 5.2% of the total variation (F(2, 27) = 5.113, p = 0.0131). The treatment groups had an even stronger effect, explaining 77.3% of the variation (F(2, 27) = 76.09, p < 0.001). However, there was no significant interaction between the hippocampal regions and treatment groups (F(4, 27) = 1.858, p = 0.1469). These results suggest that A1R antagonism mitigates the SD-induced neuroinflammation by reducing TLR4 + Iba1 + cells in the hippocampus. Since the microglial cells are more prone to morphological alterations due to noxious insults to the hippocampal microenvironment, we performed Sholl analysis (ImageJ software) to test the role of A1R antagonism in regulating SD-induced morphological alterations in glial cells. An immunofluorescence examination of the glial cell activation in the dorsal hippocampal niche followed by 48-hour SD was carried out. We found that 8-CPT decreased the activated microglial cell population in DG (F(2, 9) = 34.3; p < 0.01, Figure 16B), CA3 (F(2, 9) = 49.55; p < 0.01, Figure 16B), and CA1 (F(2, 9) = 43.11; p < 0.01, Figure 16B) in the SD animals. Microglia Schoenen ramification index was demonstrated to be increased in DG (F(2, 12) = 21.2; p < 0.01, Figure 16C), CA3 (F(2, 12) = 28.42; p < 0.01, Figure 16C), and CA1 (F(2, 12) = 51.41; p < 0.01, Figure 16C) of SD + CPT rats compared to SD. Microglia Soma area was calculated and was found to be decreased in DG (F(2, 12) = 40.7; p < 0.01, Figure 16D), CA3 (F(2, 12) = 107.8; p < 0.01; Figure 16D), and CA1 (F(2, 12) = 7.336; p < 0.05, Figure 16D) of SD + CPT rats compared to SD. We observed a decrement in endpoints per cell in DG (F(2, 12) = 8.21; p < 0.05, Figure 16E), CA3 (F(2, 12) = 8.65; p < 0.05, Figure 16E), and CA1 (F(2, 12) = 14.47; p < 0.05, Figure 16E) of SD rats as compared to CC and SD + CPT administration had no significant effect. Notably, these results demonstrate that morphological alterations in glial cells in response to SD represent the onset of neuroinflammation. These observations have contributed to a better understanding of the role of A1R in regulating SD-induced activation of Toll-like receptor 4 (TLR4) in microglial cells, instigating a cascade of neuroinflammatory pathways. 

A1R antagonism rescues the SD-induced reduction of serotonin, BDNF, and P-CREB expression 

**Figure 17:**
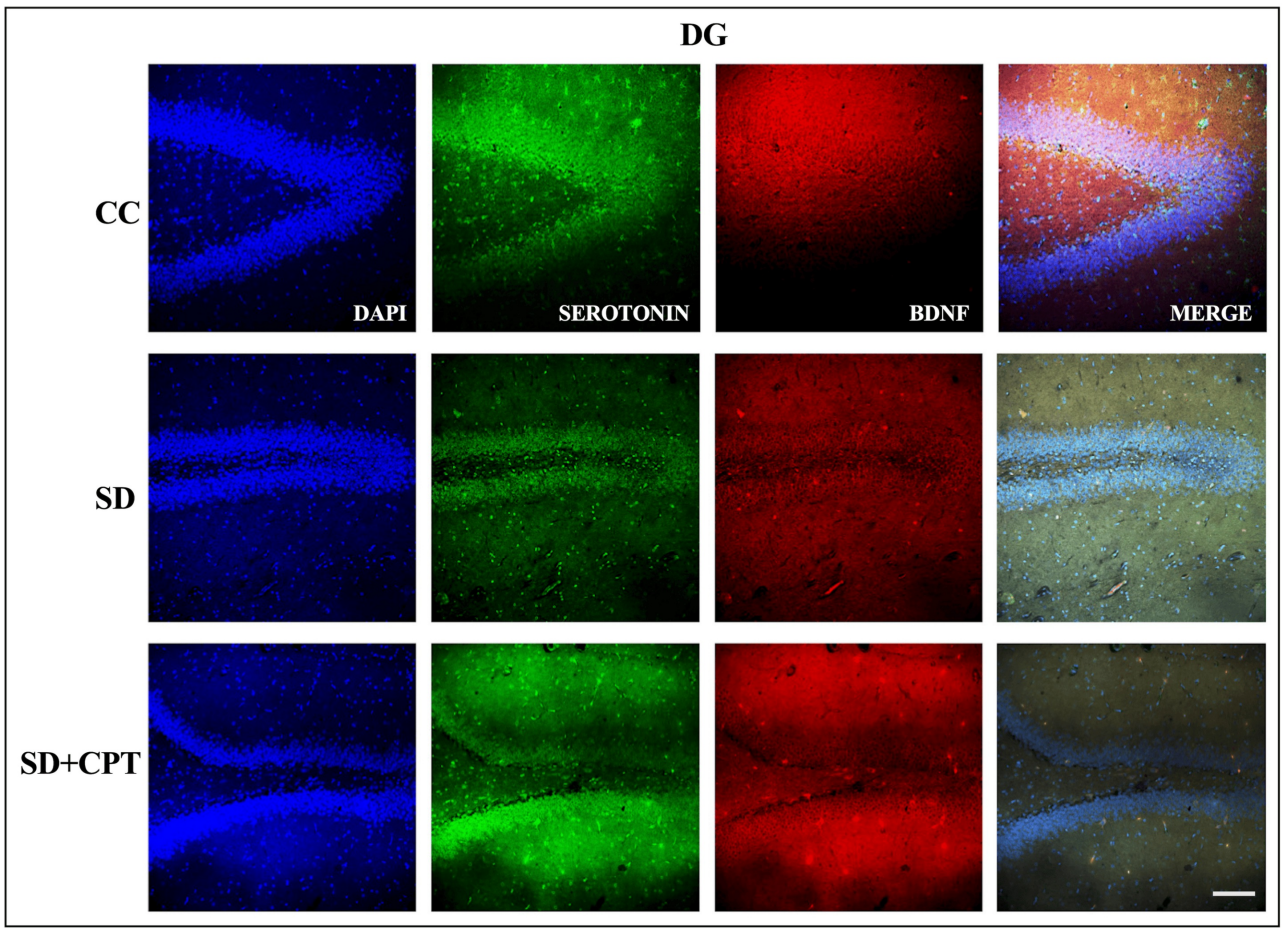
A1R regulates the expression of serotonin and BDNF in the DG region of the hippocampus following SD Representative IHC images of serotonin and BDNF expression in the DG region of hippocampus. (Mean intensity by ImageJ, scale bar, 50 μm) (one-way ANOVA, Tukey’s post-hoc test, n = 5). A1R: adenosine A1 receptor, BDNF: brain-derived neurotrophic factor, DG: dentate gyrus, SD: sleep deprivation, IHC: immunohistochemistry

**Figure 18:**
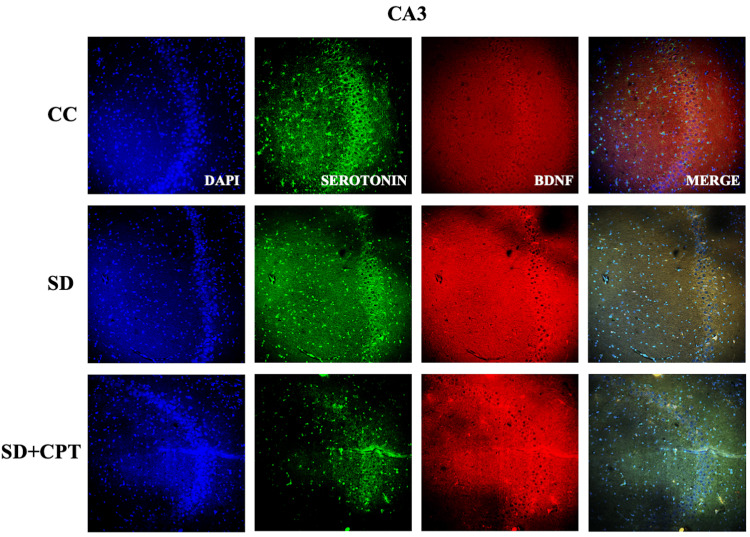
A1R regulates expression of serotonin and BDNF in the CA3 region of the hippocampus following SD Representative IHC images of serotonin and BDNF expression in the CA3 region. (Mean intensity by ImageJ, scale bar, 50 μm) (one-way ANOVA, Tukey’s post-hoc test, n = 5). A1R: adenosine A1 receptor, BDNF: brain-derived neurotrophic factor, SD: sleep deprivation, IHC: immunohistochemistry

**Figure 19:**
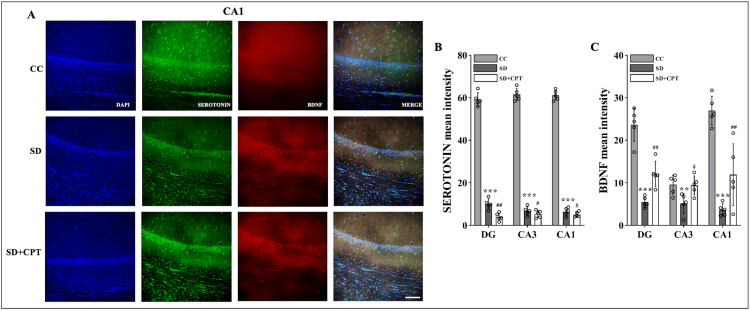
A1R regulates the expression of serotonin and BDNF in the CA1 region of the hippocampus following SD A. Representative IHC images of serotonin and BDNF expression in the CA1 region of the hippocampus. B. Statistical analysis of serotonin expression in the DG, CA3, and CA1 regions. (Mean intensity by ImageJ, scale bar, 50 μm) (one-way ANOVA, Tukey’s post-hoc test, n = 5). C. Statistical analysis of BDNF expression in the DG, CA3, and CA1 regions. (Mean intensity by ImageJ, scale bar, 50 μm) (one-way ANOVA, Tukey’s post-hoc test, n = 5) A1R: adenosine A1 receptor, BDNF: brain-derived neurotrophic factor, SD: sleep deprivation, IHC: immunohistochemistry

Although it is well studied that serotonin, BDNF, and p-CREB play a pivotal role in the neurogenesis and the consolidation of aversive memories, we sought to further investigate the role of A1R in SD-induced deficits in serotonin, BDNF, and P-CREB expression. Immunofluorescence quantification was carried out for serotonin, BDNF, and p-CREB in the hippocampal niche. We found a sharp decline in the expression of serotonin in DG (F(2, 9) = 1452; p < 0.001, Figures 17, 19B), CA3 (F(2, 9) = 1360; p < 0.001, Figures 18, 19B), and CA1 (F(2, 9) = 2015; p < 0.001, Figures 19A, 19B) following SD in comparison to cage control rats. An interesting observation in this analysis is that 8-CPT administration did not rescue the SD-induced decline of serotonin in the hippocampus (p < 0.5; Figure 19B). However, A1R antagonism increased the expression of BDNF in DG (F(2, 9) = 52.39; p < 0.01, Figure 17A, 19C), CA3 (F(2, 9) = 3.439; p < 0.5, Figures 18, 19C), and CA1 (F(2, 9) = 39.09; p < 0.001, Figures 19A, 19C) following SD. 

**Figure 20:**
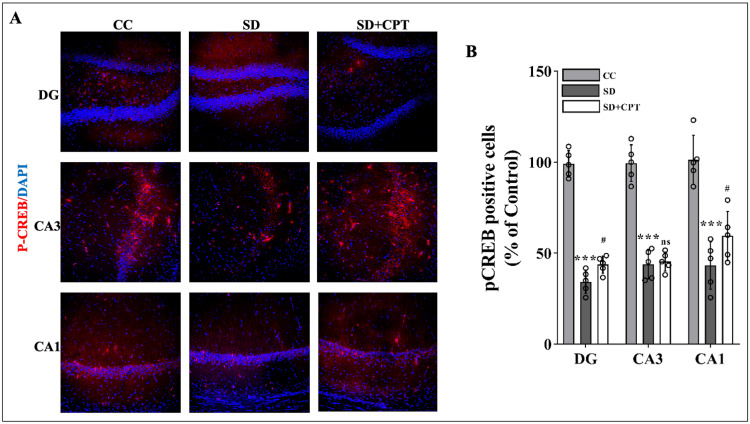
A1R regulates the P-CREB expression during SD in the hippocampus A. Representative IHC images (g) statistical analysis of p-CREB expression in the DG (F(2, 9) = 117.8; p < 0.001), CA3 (F(2, 9) = 58.74; p < 0.01), and CA1 (F(2, 9) = 17.32; p < 0.01) regions. (Mean intensity by ImageJ, white scale bar, 50 μm) (one-way ANOVA, Tukey’s post-hoc test, n = 5) A1R: adenosine A1 receptor, BDNF: brain-derived neurotrophic factor,  P-CREB: phosphorylated cAMP-response element binding protein, SD: sleep deprivation, IHC: immunohistochemistry

A diminished number of p-CREB-positive cells was observed in DG (F(2, 9) = 117.8; p < 0.001, Figures 20A, 20B), CA3 (F(2, 9) = 58.74; p < 0.01, Figures 20A, 20B), and CA1 (F(2, 9) = 17.32; p < 0.01, Figures 20A, 20B) after SD. These attenuated levels of p-CREB-positive cells were rescued only in CA1 (p < 0.01), whereas no significant improvement in the number of p-CREB-positive cells was observed in the CA3 (p = 0.5) and DG (p = 0.5) regions. These findings suggest that antagonism of A1R re-establishes the levels of BDNF and p-CREB comparable to control animals, which may have ameliorated SD-induced deficits in cued fear extinction memory recall. 

A1R antagonism prevents SD-induced upregulation of the caspase-3 and P-p38 MAPK pathway 

**Figure 21:**
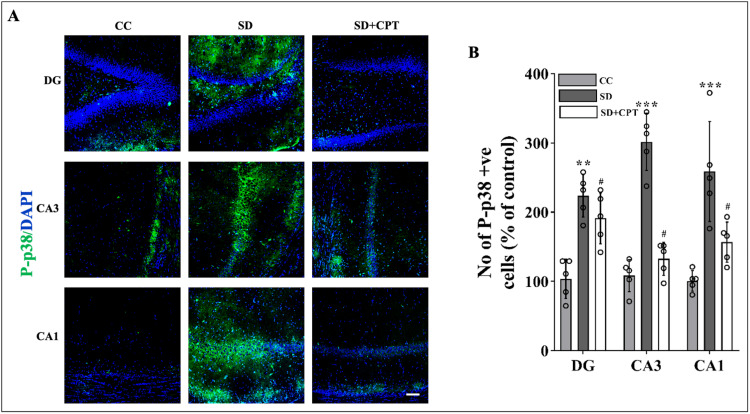
A1R antagonism reverts neuronal death in the dorsal hippocampal niche during SD A. Representative IHC images. B. Statistical comparison of P-p38 cells in the DG (F(2, 9) = 8.754; p < 0.001), CA3 (F(2, 9) = 9.644; p < 0.01), and CA1 (F(2, 9) = 4.535; p < 0.05) regions. (Mean intensity by ImageJ, scale bar, 50 μm) (one-way ANOVA, Tukey’s post-hoc test, n = 5) A1R: adenosine A1 receptor, SD: sleep deprivation, IHC: immunohistochemistry

**Figure 22:**
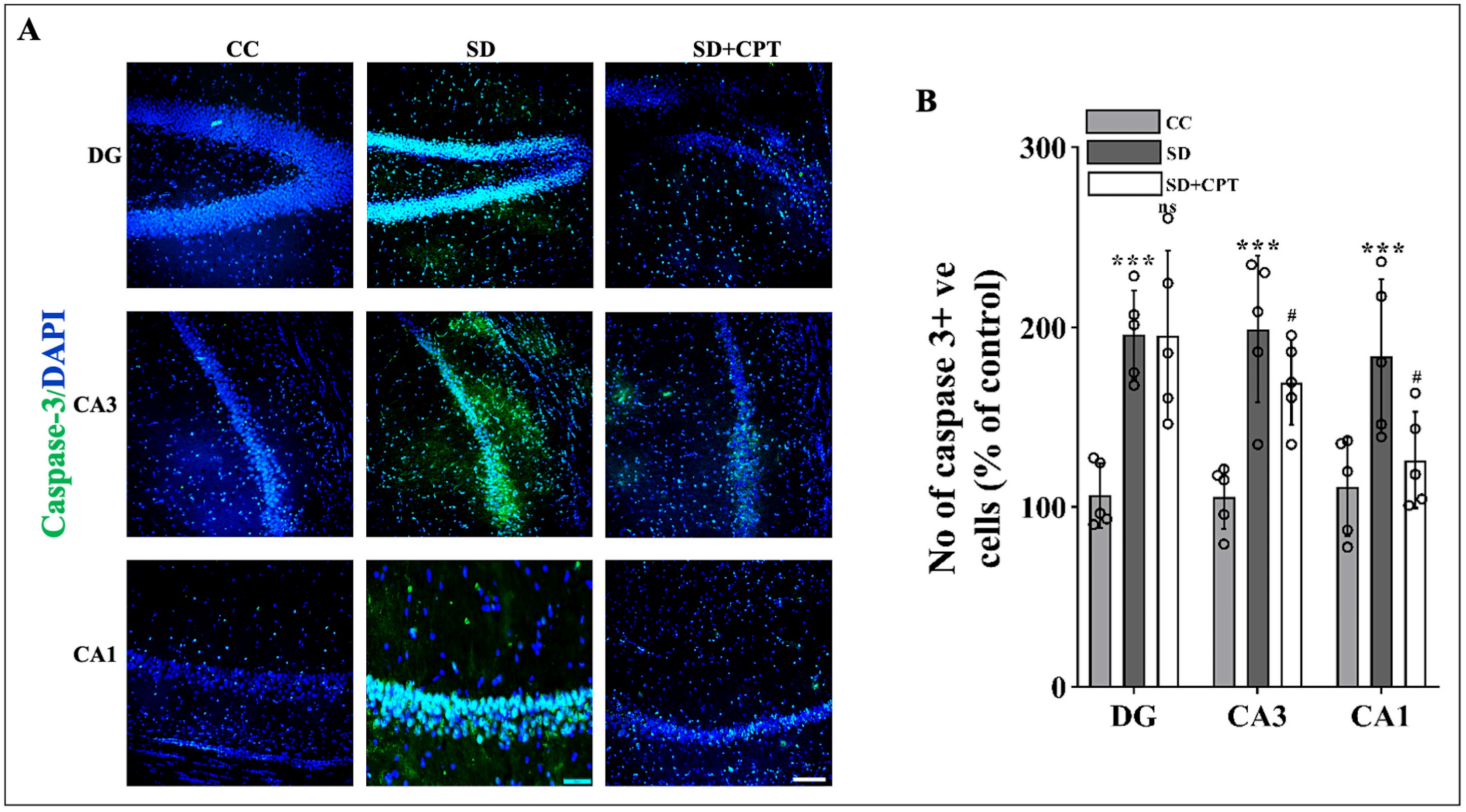
A1R antagonism reverts neuronal death in the dorsal hippocampal niche during SD A. Representative IHC images. B. Statistical comparison of caspase-3-positive cells in the DG (F(2, 9) = 10.3; p < 0.01), CA3 (F(2, 9) = 40.14; p < 0.01), and CA1 (F(2, 9) = 118.6; p < 0.01) regions. (Mean intensity by ImageJ, white scale bar, 50 μm) (one-way ANOVA, Tukey’s post-hoc test, n = 5) CC: cage control; SD: 48-hour sleep deprivation; SD + CPT: 48-hour sleep deprivation + 8-cyclopentyltheophylline, A1R: adenosine A1 receptor, SD: sleep deprivation, IHC: immunohistochemistry

Total SD hastens the progression of neuronal apoptosis that may eventuate in neurodegeneration. The number of neurons progressing towards apoptosis in the hippocampal niche is a predominant factor in regulating neurological homeostasis influencing memory consolidation and aversive processing. Since it is evident from several studies that the activation of the P-p38 MAPK pathway leads to cellular apoptosis, we investigated the neuronal loss in response to 48-hour SD in the dorsal hippocampal niche that might have paralleled the deficits in the extinction memory recall (Figure 4B) and examined the restorative potential of adenosine A1R antagonism. As shown in Figure 21B, a statistically significant increase in the number of P-p38 cells in DG (F(2, 9) = 10.3; p < 0.01, Figure 21A, 21B), CA3 (F(2, 9) = 40.14; p < 0.01, Figure 21A, 21B), and CA1 (F(2, 9) = 118.6; p < 0.01, Figure 21A, 21B) was observed in SD as compared to CC. In immunofluorescence quantification, we also observed a significant elevation in the number of activated caspase-3 positive cells CA1 (F(2, 9) = 4.535; p < 0.05, Figure 22A, 22B), CA3 (F(2, 9) = 9.644; p < 0.01, Figure 22A, 22B), and DG (F(2, 9) = 8.754; p < 0.001, Figure 22A, 22B) in sleep-deprived rats as compared to control animals. However, the antagonism of A1R during SD resulted in the restoration of caspase-3-positive cells closer to basal levels only in CA3 (p < 0.001; Figure 22A, 22B) and CA1 (Figure 22A, 22B), but not DG (Figure 22A, 22B), the reason for this effect remains unknown. In the case of P-p38-positive cells, 8-CPT administration reduced the number of P-p38-positive cells in CA1 (Figure 21A, 21B), CA3 (Figure 21A, 20B), as well as DG (Figure 21A, 21B). These results illustrate that the antagonism of A1R regulates SD-induced neuronal apoptosis by reducing the expression of P-p38 and caspase-3. 

Enhanced REM sleep with adenosine A1R antagonism during rebound sleep: differential impact on NREM sleep

While the scientific literature firmly supports the role of A1R in regulating sleep pressure homeostasis, conflicting findings concurrently exist. Numerous studies have documented that an increase in the density of A1Rs within the brain reduces sleep pressure and enhances cognitive performance [14]. An intriguing study utilizing A1R receptor knockout mice to examine the impact of SD and the blockade of A1R on sleep architecture found no significant differences in sleep patterns when compared to wild-type mice [22].

**Figure 23:**
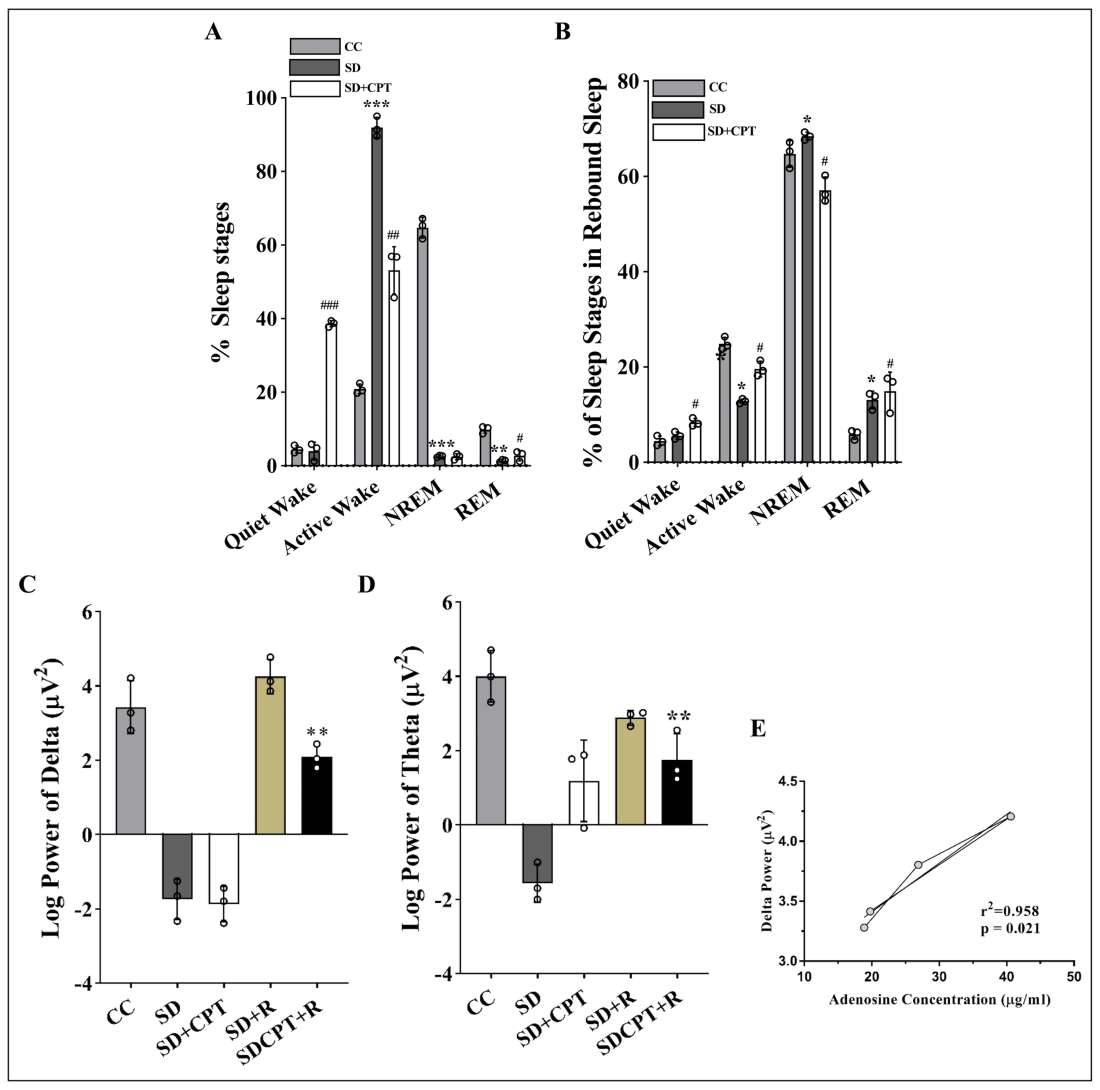
Systemic administration of 8-CPT-induced increment in REM sleep and theta power during SD A. Statistical comparison of the percentage of sleep stages in the SD and SD + CPT groups relative to the control animals with ad libitum sleep (two-way ANOVA, Tukey’s post-hoc test, n = 3). B. Statistical comparison of the percentage of sleep stages during rebound sleep in the SD and SD + CPT groups relative to control animals with ad libitum sleep revealed that NREM sleep did not increase significantly in the SD + CPT group, whereas REM sleep increased (two-way ANOVA, Tukey’s post-hoc test, n = 3). Statistical comparison of C. delta and D. theta power during rebound sleep relative to baseline sleep. (SD-R: rebound sleep post 48-hour SD; SDCPT-R: rebound sleep post 48-hour SD with the administration of 8-CPT) (F(2, 6) = 177.7, p < 0.01) (two-way ANOVA, Tukey’s post-hoc test, n = 3). E. Correlation between delta power and adenosine concentrations in SD + CPT rats (r2 = 0.958). 8-CPT: 8-cyclopentyltheophylline, SD: sleep deprivation, NREM: non-rapid eye movement, REM: rapid eye movement

**Figure 24:**
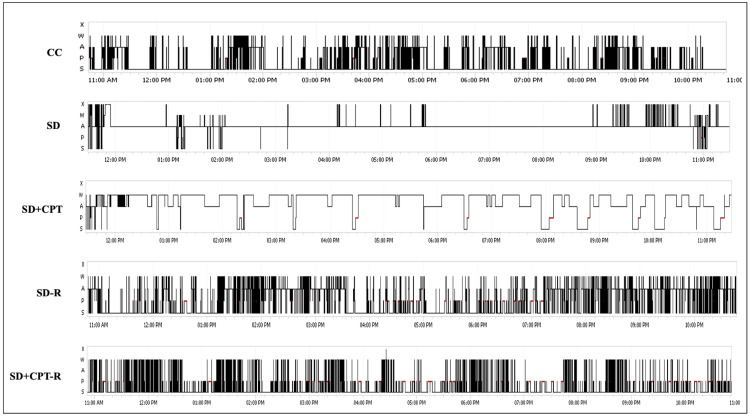
Representative hypnograms of sleep stages in all experimental groups CC: cage control; SD: sleep deprivation; NREM: non-rapid eye movement; REM: rapid eye movement; SD-R: rebound sleep group post SD; SD + CPT-R: rebound sleep post 8-CPT-treated SD animals. X: artifacts; W: quiet wake; A: active wake; P: paradoxical sleep (rapid eye movement sleep); S: non-rapid eye movement sleep

Having demonstrated that the blockade of endogenous adenosine, acting at A1Rs, results in increased microglial ramification and thereby alleviates neuroinflammation caused by SD, we proceeded to investigate whether A1R antagonism modulates sleep architecture during SD and subsequent rebound sleep. The results from the two-way repeated-measures ANOVA showed a significant increase in REM sleep following the systemic administration of 8-CPT during SD (#p < 0.05; Figure 23A), whereas no significant change was observed in NREM sleep (F(3, 24) = 1620; Figure 23A) as compared to SD rats with vehicle administration. In addition, it is clear from our observations that A1R antagonism significantly increased the duration of the quiet wake stage in the polysomnographic recordings (F(2, 6) = 177.7, p < 0.01). Figure 23B demonstrates that both total NREM and REM sleep increased during rebound sleep in SD rats treated with the vehicle (F(3, 8) = 1550, p < 0.05). However, A1R antagonism did not result in an increase in NREM sleep. Interestingly, the REM sleep stage was significantly increased during rebound sleep (F(8, 16) = 1.362, p < 0.05; Figure 23B). Throughout the 48-hour SD period, our power spectral analysis revealed a significant decrease in EEG delta power (Figure 23C). Notably, our findings illustrate that the administration of the A1R antagonist similarly led to a reduction in EEG delta power, but the extent of this reduction was not significantly different from the reduction observed in the SD rats (Figure 23C). This observation supports the equivalent reduction in NREM sleep in both SD and SD + CPT rats. Distinctly heightened EEG delta power was evident during the rebound sleep in the SD rats compared to the group treated with 8-CPT (SDCPT-R) (F(2, 6) = 12.87, p < 0.01, Figure 23C). Figure 23D demonstrates a substantial decrease in EEG theta power during SD compared to control animals and notably depicts a marked increase in EEG theta power induced by A1R antagonism in comparison to the SD group (F(2, 6) = 101.6, p < 0.001). Nevertheless, the A1R antagonism did not lead to an increase in EEG theta power compared to the SD rats during the rebound sleep following SD (F(2, 6) = 6.686, p = 0.02; Figure 23D). The observed positive correlation between delta power and adenosine concentration implies an association between elevated extracellular adenosine levels during SD and the subsequent heightened delta power during rebound sleep (p < 0.05; r2 = 0.95; Figure 23E). The delta and theta power during the 48-hour rebound sleep following SD in CPT-administered rats did not exhibit complete recovery in comparison to SD rats. Figure 24 shows the representative sleep architecture of all experimental groups illustrated by their corresponding hypnograms stating the sleep scoring stages such as quiet wake (W), active wake (A), paradoxical sleep (P), NREM sleep (S) and movement artifacts or noise (X). Future studies can focus on understanding the specific mechanisms through which A1R antagonism affects EEG power, particularly in relation to sleep stages. 

Therefore, these findings indicate a potential association between adenosine dynamics, A1R modulation, and altered EEG powers during sleep states. Addressing these facets in future studies could offer deeper insights into the mechanisms governing sleep architecture and aid in the development of targeted interventions to optimize sleep quality and recovery. The significant increase in REM sleep following systemic administration of 8-CPT during SD presents an exciting area for further research to understand the specific mechanisms influencing REM sleep regulation upon A1R antagonism. Consequently, further investigations targeting the specific effects of A1R antagonism on sleep stages during rebound sleep, elucidating the intricacies of REM sleep regulation, and exploring optimal interventions for sleep architecture improvement post-SD are pivotal to advance our understanding of sleep neurobiology and potential therapeutic avenues.

## Discussion

In this study, we explored perturbations elicited by 48-hour SD on fear extinction memory recall and dynamics of deteriorations in neuronal mechanisms in the dorsal hippocampus. In addition, we explored the efficacy of 8-CPT, an adenosine A1R antagonist, in mitigating these dysregulations. SD significantly impacts cognitive functions, particularly memory consolidation and emotional processing. In our study, 48 hours of SD post-extinction training led to impaired fear extinction memory recall, evidenced by elevated freezing scores. Administration of an adenosine A1R antagonist ameliorated these deficits, indicating A1R's crucial role in the hippocampus. While fear extinction memory is consolidated in the amygdala and regulated by the prefrontal cortex, the hippocampus, particularly the dentate gyrus, interacts with other brain regions to associate contextual profiling of fear extinction and is integral to memory retrieval. Our findings align with studies showing that SD alters the hippocampal microenvironment, disrupting fear extinction memory consolidation [23]. Multiple studies have revealed that SD-induced deficits in fear extinction memory consolidation could be due to the alteration in the microenvironment of the dorsal hippocampus [24]. Next, we found an increase in A1R expression during SD suggests a compensatory mechanism against the detrimental effects of sleep loss on synaptic plasticity and memory recall. Further increase in A1R expression during antagonist administration implies a complex feedback loop modulating adenosine signalling, sleep homeostasis, and memory processes. A1R activation is neuroprotective when agonists are administered before noxious stimuli, but desensitization occurs with prolonged exposure, leading to neuronal damage [25]. This loss of efficiency seems to result from desensitization of A1R after prolonged exposure to adenosine.

In our study, we examined the possibility of A1R antagonism exerting a neuroprotective effect since A1R agonism leads to desensitization and paradoxical neuronal damage during prolonged noxious insults to the central nervous system. Future research should explore the role of other brain regions and their interactions with the hippocampus. SD is known to induce significant cognitive deficits and impair memory consolidation processes by disrupting synaptic plasticity mechanisms [26]. Recent studies employing synaptic proteome analysis reveal both upregulation and downregulation of proteins involved in synaptic transmission, assembly, and other neuronal processes. Adenosine, acting through its receptors, plays a crucial role in regulating synaptic plasticity, particularly in modulating excitatory glutamatergic synaptic transmission. Prolonged activation of adenosine A1Rs during conditions such as hypoxia has been shown to induce the endocytosis of AMPA receptors, leading to synaptic downscaling and impaired long-term potentiation (LTP). Contrarily, elevated A1R availability in humans represents increased resilience to the adverse effects of SD, as compared to individuals with lower A1R availability [14]. These contrasting results prompted us to focus more on A1R in the hippocampus during SD. Previously, we demonstrated that 48 hours of SD resulted in the downregulation of synaptic plasticity proteins, including synaptophysin, synapsin, and PSD-95, and that this downregulation was attenuated by caffeine and modafinil [27]. Building upon these findings, our results demonstrated a significant downregulation of synaptic plasticity (PSD-95 and synaptophysin) in the dorsal hippocampus following SD, which was mitigated by the antagonism of A1R. We hypothesized that the inhibition of prolonged A1R sensitization during SD may have prevented the internalization of AMPA receptors, thereby promoting synaptic upscaling. The precise elucidation of molecular mechanisms and signalling pathways through which CPT manifests its effects on these synaptic proteins remains an area yet to be comprehensively addressed. 

Our current investigation has revealed that SD significantly elevates the expression of pro-inflammatory cytokines and alters microglial activity in the hippocampus, aligning with previous findings on neuroinflammatory responses to sleep loss [12]. These alterations were mitigated by the antagonism of the A1R, as evidenced by immunostaining. Specifically, our RT-PCR analysis demonstrated a marked increase in the mRNA levels of pro-inflammatory cytokines, such as IL-1α, IL-1β, IL-1r, IL-1a, IL-2rα, IL-2rβ, TNF-α, and IL-6, during SD. Concurrently, anti-inflammatory cytokines, including IL-1ra, IL-4, IL-10, IL-11, and IL-13, were downregulated. Crucially, A1R antagonism attenuated the expression of pro-inflammatory cytokines and augmented anti-inflammatory cytokine levels within the hippocampus. To further our understanding, we conducted a functional analysis of differentially expressed cytokine genes using Gene Ontology (GO) enrichment in Cytoscape. The analysis of BPs, CCs, and MF revealed significant enrichment in immune system processes, chemotaxis, responses to various stimuli, extracellular compartments, particularly the extracellular space and plasma membrane components, cytokine activity, protein binding, and receptor binding.

Further research is needed to investigate the long-term impact of A1R antagonism on neuroinflammation and cognitive function. Having established the role of A1R and its inhibition in regulating fear memory recall, synaptic plasticity, and neuroinflammation during SD, we investigated whether A1R antagonism is involved in the regulation of TLR4-mediated microglial activation during SD. Recent studies have highlighted the function of TLR4 as a mediator of cellular pro-inflammatory responses triggered by SD and other neurological disorders [28]. The present study shows that A1R antagonism downregulates TLR4 expression, suggesting a regulatory role of adenosine in the immune response during SD. Our findings indicate that A1R antagonism downscales TLR4 upregulation during SD, evidenced by the decrease in TLR4 + Iba + cells, which were elevated during SD. We speculate that induction of TLR4 in activated microglia may disrupt Ca2+ homeostasis and cause neurodegeneration. This is corroborated by our further observations of increased p-P38- and caspase-3-positive cells. In our prior investigations, we have substantiated the presence of neurodegeneration in both the amygdala and hippocampus following exposure to hypobaric hypoxia (HH) [18,29] and impaired memory consolidation specifically in the CA1 region of the hippocampus. Another possibility could be that A1R antagonism-mediated Ca2+ homeostasis dysregulations during SD may potentially disrupt endogenous sleep pressure signalling via the A1R.

To our knowledge, this is the first study to show the effect of A1R antagonism on SD-induced TLR4-mediated enhanced microglial activation in the hippocampus. Our Sholl analysis revealed that the soma area of microglial cells significantly increased in the hippocampus of SD rats, with a decrease in endpoints per cell and the Schoenen ramification index, indicating a less complex and more amoeboid morphology. Administration of 8-CPT significantly attenuated the enhanced glial activation and morphological changes in microglial cells in SD rats. Future studies should emphasize longitudinal studies assessing the long-term effects of A1R antagonism on cognitive function and neuroinflammation post-SD. 

Previously, it was reported that COX-1 neurotoxicity was microglial-dependent; hence, COX-1 inhibition reverted microglia activation and rescued adult neurogenesis deficits [19]. In continuation, we examined the effects of SD on BDNF and serotonin, known to bi-directionally regulate adult neurogenesis in the hippocampus. CREB resilience to chronic stress is enhanced by the upregulation of BDNF in serotonergic neurons, facilitating adult neurogenesis in the hippocampus [30]. In addition, the activation of A2A receptors has been shown to increase BDNF expression in the brain, conferring neuroprotection. These reports led us to investigate the expression patterns of BDNF and serotonin post-SD and to assess how A1R antagonism influences their expression in the hippocampus. Our study revealed a significant decrease in the expression of BDNF and serotonin following 48-hour SD, along with a critical reduction in p-CREB expression. p-CREB directly regulates BDNF-induced gene expression and other neurotrophin-elicited neuronal activities. We found that adenosine A1R antagonism rescued p-CREB and BDNF expression but not serotonin in the hippocampus, an intriguing observation that warrants further investigation.

In addition to investigating the molecular effects of A1R antagonism in the hippocampus, we also examined its impact on sleep architecture and EEG power during rebound sleep post-SD. Previous studies have shown that adenosine A1R blockade in the prefrontal cortex reduces NREM and REM sleep states and decreases EEG delta and theta power. Another study found that A1R blockade inhibited the antidepressant effects of REM SD following stress, highlighting the role of A1R in REM sleep and emotional memory consolidation [31]. However, the effects of systemic A1R antagonist administration during SD on rebound sleep architecture in rats were unclear. Our findings indicate that A1R antagonism during SD decreases NREM sleep and relatively increases REM sleep compared to sleep-deprived rats. These results, while surprising, may be explained by two potential mechanisms. First, systemic A1R antagonist administration during SD may trigger a compensatory mechanism of homeostatic sleep regulation, specifically improving REM sleep and also during rebound sleep. We also speculate that parallel sleep-wake regulatory systems, such as noradrenergic and serotonergic activity, play a role in this process. Second, while adenosine's sleep-inducing effects are primarily mediated by A1R, other adenosine receptor subtypes may become more relevant during SD. The inhibitory action of A1R on cholinergic neurons in wake-promoting brain regions induces sleep, but A1R antagonism may simultaneously activate GABAergic neurons in the basal forebrain, enhancing REM sleep during SD. We also speculate that blocking inhibitory A1R may lead to compensatory increases in the expression and binding affinity of other adenosine receptor subtypes, improving sleep during the SD procedure. However, systemic A1R antagonist administration could have peripheral effects during SD that are not yet fully understood. Despite our findings, we acknowledge data suggesting that blocking adenosine receptors reduces sleep, indicating the need for further investigation into adenosine receptor gene expression patterns during SD.

The present study elucidates the complex interplay between SD, hippocampal neurobiology, and fear extinction memory recall in rats. We found that 48-hour SD significantly disrupts fear extinction memory, linked to alterations in the hippocampus. This disruption is associated with dysregulation of synaptic plasticity and BDNF, serotonin, and p-CREB, leading to impaired neurogenesis. Furthermore, SD-induced neuroinflammation is characterized by TLR4 upregulation and glial activation. Notably, A1R antagonism during SD mitigated these detrimental effects, restoring fear extinction memory and reducing neuroinflammation and glial activation. In addition, A1R antagonism increased REM sleep without significantly affecting NREM sleep. However, the incomplete recovery of delta and theta power during rebound sleep indicates the need for further research. Future investigations could concentrate on exploring the precise relationship between adenosine fluctuations and specific EEG power spectral densities. In addition, understanding the rationale of delta and theta power in CPT-administered rats not fully recovering during 48-hour rebound sleep following SD might be crucial for developing interventions to enhance sleep recovery. These findings highlight the potential of targeting adenosinergic signalling through A1R antagonism as a therapeutic strategy to ameliorate SD-induced cognitive impairments. Further studies are required to understand the long-term implications and molecular mechanisms underlying these effects.

Limitations

Although the present study showed strong evidence of the role of the adenosine A1R in SD-induced neurological deficits, several limitations still need to be addressed. First, the effects of A1R antagonism on neuroinflammation, sleep architecture, and memory recall were assessed only for a duration of 48 hours of SD. Second, although dosage optimization of the A1R antagonist was previously reported by our group, we adopted a single dosage of 8-CPT. Different dosages and zeitgeber timings for antagonist administrations could have been explored. Third, systemic administration of 8-CPT might have affected cardiovascular and immune systems, which may have affected our interpretations of neuroinflammatory and behavioural results. Fourth, for a comprehensive understanding of the ameliorating effects of A1R antagonism, generating longitudinal data on the role of A1R in varying durations of SD is also imperative. Fifth, it is well known that the prefrontal cortex and amygdala play critical roles in fear extinction; however, our experimental data focussed on the hippocampus since its neuronal microenvironment is underexplored in the context of SD and acts as a primary site of memory consolidation and retrieval. Sixth, we focused only on the adenosine A1R subtype, although other subtypes of adenosine receptors including A2A, A3, and A4 play critical roles in sleep homeostasis. In our experiments, the relatively small sample size of five per group for biochemical analyses was implemented; however, based on the sample size calculations and desirable statistical power in G* Power, five animals in each group for immunohistochemical and ELISA quantification and 11 animals in each group for behavioural analyses was sufficient for achieving the statistical power of 80%.

Despite all these promising results on the inhibition of A1R receptors by 8-CPT, it is imperative to consider the detrimental side effects of these xanthine analogue drugs. The negative consequences of 8-CPT have been reported to elicit respiratory stimulant effects, increase cardiovascular stress and irritability, and burn calories unnecessarily leading to the inhibition of growth, and in worst cases, it could lead to tachycardia. However, these drugs are predominantly used in the therapeutic interventions of sleep apnoea and asthma patients as bronchodilators. However, it is extremely important to consider these cardiovascular and respiratory side effects of xanthine analogue molecules. Although all these limitations exist, to the best of our knowledge, our study is the first of its kind to assess the role of A1R in SD-induced neuroinflammation and extinction memory recall in rats evidenced by sleep architecture and immunohistochemical analyses.

## Conclusions

The present study demonstrates that blockade of adenosine A1R signalling during 48-hour SD significantly improves fear extinction memory recall, synaptic plasticity, and anti-inflammatory cytokines and attenuates anxious-depressive-like behaviour and TLR-4-mediated microglial activation in the hippocampus. Intriguingly, it also enhanced REM sleep without significantly affecting NREM sleep. Current experimental evidence generated in rats could pave the way for further validation studies addressing the translational potential of these A1R antagonists in humans, carefully considering their off-target interactions, including cardiovascular and respiratory effects. These findings highlight the potential of targeting adenosinergic signalling, particularly A1R as a therapeutic target to ameliorate SD-induced cognitive impairments.
